# GLP-1 as a Regulator of Oxidative Signalling in Metabolic Health and Disease

**DOI:** 10.3390/antiox15101283

**Published:** 2026-10-03

**Authors:** Natalia Kurhaluk, Renata Kołodziejska, Halina Tkaczenko

**Affiliations:** 1 Institute of Biology, Pomeranian University in Słupsk, Arciszewski St. 22B, 76-200 Słupsk, Poland; 2 Department of Medical Biology and Biochemistry, Faculty of Medicine, Collegium Medicum in Bydgoszcz, Nicolaus Copernicus University in Toruń, Karłowicz St. 24, 85-092 Bydgoszcz, Poland; renatak@cm.umk.pl

**Keywords:** incretin-driven redox modulation, mitochondrial resilience pathways, oxidative stress biomarkers, microbiota–incretin interactions, nutritional redox regulators, metabolic cytoprotection

## Abstract

Glucagon-like peptide-1 (GLP-1) has emerged as a key regulator of metabolic, inflammatory and oxidative homeostasis, extending beyond its traditional function as an incretin hormone. This narrative review summarises the latest evidence from two decades of research into the role of GLP-1 in redox biology and metabolic regulation in tissues affected by obesity, type 2 diabetes and metabolic dysfunction-associated steatotic liver disease. There is growing evidence that GLP-1 signalling modulates multiple oxidative pathways, including AMPK-dependent metabolic reprogramming, mitochondrial quality control, nitric oxide–cGMP signalling, the regulation of ferroptosis, and the suppression of inflammatory pathways such as the NLRP3 inflammasome. Through these mechanisms, GLP-1 contributes to maintaining cellular redox balance, mitochondrial resilience, and metabolic adaptation in conditions of chronic oxidative stress. The review also emphasises the complementary function of GLP-1 receptor agonists (GLP-1RAs) and natural bioactive compounds such as polyphenols, anthocyanins, terpenoids and omega-3 fatty acids. These compounds may reinforce antioxidant defences, reduce inflammatory signalling and support mitochondrial function. Particular attention is given to the role of the gut microbiota in determining GLP-1 responsiveness. Gut microbiota-derived metabolites, including short-chain fatty acids (SCFAs) and bile acid derivatives, contribute to incretin secretion, intestinal barrier maintenance, and systemic redox regulation. This review integrates endocrinology, oxidative stress biology, microbiome science and nutritional biochemistry to provide a comprehensive framework describing GLP-1 as a multi-organ metabolic signal that coordinates endocrine, immune, mitochondrial and redox pathways. These insights will inform the development of future therapeutic strategies that combine GLP-1-based pharmacology with precision nutrition and microbiome-targeted approaches, with the aim of improving metabolic resilience and addressing the oxidative dysregulation associated with chronic metabolic diseases.

## 1. Introduction

Metabolic disorders such as obesity, insulin resistance, metabolic syndrome and type 2 diabetes mellitus (T2DM) pose significant global health challenges. These disorders are closely associated with cardiovascular disease, metabolic dysfunction-associated steatotic liver disease (MASLD), metabolic dysfunction-associated steatohepatitis (MASH), chronic kidney disease and neurodegenerative complications [1,2]. The shift in terminology from NAFLD/NASH to MASLD/MASH highlights the pivotal role of metabolic dysfunction, oxidative stress, and impaired cellular resilience in the development of steatotic liver disease. Despite their diverse clinical presentations, these conditions share common biological features, including oxidative stress, mitochondrial dysfunction, chronic low-grade inflammation and reduced metabolic flexibility, forming a common pathological foundation [3,4,5].

Disruption of redox homeostasis occurs early in metabolic deterioration and progressively shapes cellular responses to nutrient excess [4,5,6]. Lifestyle interventions, such as adherence to a Mediterranean diet, can improve metabolic parameters and partially restore oxidative balance [7,8,9], yet persistent redox instability and incomplete recovery of mitochondrial function point to deeper alterations in cellular signalling and organelle quality control [10,11,12]. These early, redox-driven changes provide a natural entry point for examining how GLP-1-related pathways intersect with mitochondrial adaptation and metabolic regulation in disease states. Various nutritional and pharmacological strategies have been investigated to restore redox homeostasis. These include caloric restriction, antioxidant supplementation, dietary bioactive compounds and the modulation of advanced glycation end products [13,14,15,16,17,18,19,20,21,22]. However, these interventions usually target individual components of metabolic dysfunction, failing to explain how nutrient sensing, mitochondrial activity, oxidative signalling and endocrine regulation are integrated at a molecular level.

Among the endogenous regulators of metabolic adaptation, glucagon-like peptide-1 (GLP-1) has emerged as a pivotal signalling molecule linking nutrient availability to mitochondrial and redox responses. Initially described as a glucose-dependent insulinotropic hormone, GLP-1 is now recognised as a multifunctional regulator influencing mitochondrial function, endothelial integrity, inflammatory pathways, lipid metabolism, autophagy and antioxidant defences [23,24]. Activation of the GLP-1 receptor improves vascular function, reduces oxidative stress, preserves mitochondrial integrity, and protects metabolically vulnerable organs [25,26,27,28]. Dietary composition and microbiota-derived signals also influence GLP-1 biology. High-protein diets have been shown to enhance incretin secretion and increase circulating GLP-1 levels [29]. Similarly, *Lactobacillus reuteri* supplementation improves incretin responses and insulin secretion in humans [30]. Additionally, plant-derived bioactive compounds, including anthocyanins and phytochemicals with GLP-1 receptor-modulating activity, may influence GLP-1-dependent metabolic and redox pathways [31,32,33]. The sensing of nutrients by intestinal L-cells further supports the close relationship between dietary signals and GLP-1 secretion [34]. Taken together, these findings establish GLP-1 as a molecular interface that connects nutrition, microbiota, mitochondrial biology and redox signalling.

This review provides an integrated framework that connects these domains through the following conceptual model: diet/microbiota → GLP-1 signalling → mitochondrial and redox pathways → metabolic signalling → tissue outcomes. The existing literature has typically examined oxidative stress, incretin physiology, mitochondrial dysfunction and natural product modulation in isolation, which is an approach that is in need of improvement. In contrast, this review examines the potential links between these interconnected fields, including GLP-1 signalling, nutrient- and microbiota-derived signals, mitochondrial function, redox homeostasis, and metabolic regulation. Particular attention is given to the bidirectional relationship between GLP-1 signalling and oxidative stress, considering both the potential influence of redox disturbances on GLP-1-producing cells and the reported effects of GLP-1 signalling on mitochondrial, antioxidant, and inflammatory pathways. The review outlines the relationship between dietary factors, gut microbiota, intestinal L-cells, and GLP-1 secretion. It then examines oxidative stress and mitochondrial dysfunction as interconnected features of metabolic disorders, followed by a focused analysis of the reciprocal interactions between GLP-1 signalling and redox pathways. Evidence from various tissues and pharmacological approaches is considered to elucidate the contribution of these interactions to metabolic and tissue outcomes. The final sections discuss the implications of this integrated framework and highlight areas that require further research.

## 2. Methodology

This narrative review aimed to provide a critical synthesis of current knowledge regarding the interplay between oxidative signalling, metabolic dysfunction, and GLP-1 biology. Unlike systematic reviews, which are designed for quantitative data aggregation, a narrative approach was considered appropriate because the available evidence encompasses diverse experimental models, clinical populations, dietary strategies, lifestyle interventions, and pharmacological approaches targeting incretin pathways. Such an interpretative framework allows mechanistic findings to be considered alongside emerging evidence from clinical research, while also accommodating the considerable heterogeneity of the available literature.

A comprehensive literature search was performed using major biomedical databases, including PubMed, Scopus, and Web of Science, which offer broad coverage of clinical research, metabolic studies, and investigations into oxidative and mitochondrial biology. The search primarily covered publications from the last ten years to capture recent advances in GLP-1 receptor agonist therapies, current approaches to metabolic syndrome and type 2 diabetes management, and advances in understanding mitochondrial dysfunction and redox regulation. However, seminal publications that established the fundamental concepts of GLP-1 physiology, incretin signalling, and oxidative stress mechanisms were also consulted when necessary to provide the biological background for interpreting more recent findings.

The search strategy was designed to identify studies addressing the complex relationship between GLP-1 signalling and metabolic redox homeostasis. Both controlled vocabulary and free-text terms were used, including GLP-1, GLP-1RAs, incretins, oxidative stress, reactive oxygen species, mitochondrial function, metabolic syndrome, insulin resistance, type 2 diabetes, endothelial dysfunction, inflammation, antioxidants, polyphenols, exercise training, hepatic steatosis, and vascular regulation. Boolean operators were used to combine these concepts into searches such as “GLP-1 AND oxidative stress”, “metabolic syndrome AND redox regulation”, and “type 2 diabetes AND mitochondrial dysfunction”. The search was focused primarily on peer-reviewed articles published in English, with particular attention given to human studies, clinical trials, and randomised controlled trials. Additionally, the reference lists of highly relevant publications were manually examined to identify additional studies that could provide relevant evidence on the antioxidant and metabolic effects associated with GLP-1 signalling.

Studies were considered relevant if they investigated oxidative stress mechanisms, redox biomarkers, mitochondrial function, endothelial homeostasis, inflammatory signalling, or incretin-related pathways in the context of obesity, insulin resistance, metabolic syndrome, or type 2 diabetes. Particular emphasis was placed on clinical evidence evaluating the effects of GLP-1 receptor agonists, nutritional interventions, antioxidant strategies, and exercise-based approaches on metabolic and oxidative balance. Studies were excluded if they lacked practical relevance, did not address oxidative or metabolic outcomes, focused exclusively on unrelated endocrine pathways, or were limited to conference abstracts and non-peer-reviewed sources. Thus, the methodological approach was intended to integrate molecular mechanisms with clinical observations and provide a broad perspective on the contribution of GLP-1 signalling to oxidative balance in metabolic health and disease. By connecting redox biology with incretin physiology, the review also considers emerging therapeutic opportunities and highlights areas in which further investigation is warranted.

## 3. GLP-1 Biology and Metabolic Functions

The physiological concept of incretin signalling emerged from early observations in 1906 showing that intestinal extracts lowered blood glucose, a finding that was later reinforced by studies conducted during the 1960s and 1970s demonstrating that an oral glucose load elicits a greater insulin response than an intravenous glucose load at matched glycaemia. These findings established the incretin effect and stimulated subsequent efforts to identify gut-derived insulinotropic factors. Contemporary research has since clarified the mechanisms governing GLP-1 synthesis and secretion within this framework. GLP-1 is now recognised as a product of tissue-specific pro-glucagon processing in enteroendocrine L cells, forming a central link between nutrient sensing and coordinated metabolic responses. Recent findings further expand this regulatory landscape by showing that GLP-1 production is shaped not only by chemical and hormonal stimuli but also by the physical and metabolic characteristics of the intestinal environment [34]. Emerging evidence further suggests that oxidative stress contributes to intestinal barrier disruption and chronic inflammation, whereas the maintenance of intestinal redox balance and barrier integrity may support enteroendocrine signalling and metabolic regulation [35]. Proglucagon is also expressed in pancreatic α-cells and neurons of the nucleus tractus solitarius, indicating that GLP-1-related signalling extends across multiple physiological systems [36]. GLP-1 secretion is regulated by the integration of hormonal, neural, nutrient-derived and local intestinal factors. Impaired L-cell responsiveness contributes to reduced circulating GLP-1 concentrations in type 2 diabetes [37].

Piezo1-dependent mechanosensation has been identified as a direct regulator of GLP-1 production in L-cells [38], while the activation of the same pathway influences hepatic lipid metabolism through GLP-1-mediated mechanisms [39]. Together, these observations position intestinal L-cells as highly specialised metabolic sensors that integrate chemical, neural, hormonal, and mechanical information. This multifactorial regulatory network provides a mechanistic basis for the adaptability of GLP-1 signalling to changes in metabolic status and its contribution to systemic homeostasis. The broad physiological effects of GLP-1 are determined by the distribution and functional properties of its receptor, GLP-1R, a class B G protein-coupled receptor involved in multiple metabolic pathways. GLP-1R mediates incretin effects related to insulin secretion, appetite regulation, gastrointestinal motility, and cardiovascular responses [40], and its expression across pancreatic β-cells, the gastrointestinal tract, cardiovascular tissues, and the central nervous system [24] enables GLP-1 to coordinate endocrine, neural, and metabolic processes that extend well beyond its classical role in glucose regulation.

The physiological actions mediated through the GLP-1R are particularly evident in the regulation of gastrointestinal function and postprandial metabolism. GLP-1 and its analogues are known to delay gastric emptying through vagal mechanisms [41], a response that contributes to postprandial glucose control and appetite suppression. This gastrointestinal modulation establishes a direct link between gut-derived signalling and systemic metabolic regulation. Meanwhile, mounting evidence suggests that GLP-1R signalling is influenced by intracellular and membrane-level regulatory processes. Sympathetic nerve activity has been shown to suppress GLP-1 release and alter brain glucose utilisation [42], revealing a neuro-enteroendocrine pathway that connects peripheral incretin signalling with central metabolic regulation and cognitive function. Complementary findings indicate that membrane microdomains also influence receptor behaviour, as evidenced by the identification of caveolin-1 as a structural regulator of GLP-1R activity [43]. Thus, these observations emphasise that the action of GLP-1 depends not only on the availability of the hormone, but also on the cellular and membrane environment in which receptor signalling occurs. This provides a mechanistic basis for the variability in incretin sensitivity across tissues.

The physiological importance of GLP-1 is most evident in its regulation of glucose metabolism, appetite, gastrointestinal function, and energy balance. Numerous experimental and clinical studies have demonstrated that GLP-1 enhances glucose-dependent insulin secretion while suppressing glucagon release, representing the central mechanism underlying its glucose-lowering effects [44,45,46,47]. Unlike insulin-independent glucose-lowering pathways, GLP-1 preserves physiological regulation by amplifying insulin secretion primarily under conditions of elevated glucose availability, thereby reducing the risk of excessive hypoglycaemia. The magnitude of endogenous GLP-1 responses is also influenced by dietary composition. Huber et al. (2024) demonstrated that dietary composition strongly influences fasting and stimulated GLP-1 secretion, thereby linking nutrient quality with incretin-mediated metabolic regulation [48].

Nutrient sensing is just one of the mechanisms that regulate GLP-1 release; intestinal microbial metabolism also plays a significant role in incretin control. Short-chain fatty acids generated through microbial fermentation stimulate GLP-1 secretion via FFAR2 activation [49], establishing a direct molecular link between microbiota-derived metabolites and enteroendocrine signalling. Complementary evidence shows that combinations of amino acids and fatty acids exert synergistic effects on GLP-1 secretion [34], indicating that nutrient-derived and microbially generated substrates converge on L-cells to amplify endogenous incretin responses. These findings suggest that GLP-1 works as a unifying messenger in the body, translating signals from food and microbes into coordinated responses in the body.

Intracellular signalling pathways add another regulatory layer by coupling cellular energy status to endocrine function. The mTOR pathway has been identified as a key regulator that connects nutrient availability with GLP-1 biosynthesis [50], and mTOR-dependent mechanisms also mediate the effects of ghrelin on GLP-1 production [51]. Collectively, these observations show that GLP-1 secretion reflects the combined influence of nutrient inputs, hormonal signals, microbial metabolites and intracellular energy-sensing pathways, rather than being driven by any single stimulus. Beyond its traditional function in regulating glucose, GLP-1 has emerged as a key regulator of inflammatory processes in metabolically active tissues, particularly adipose tissue and the liver. Growing evidence links impaired GLP-1 signalling with chronic low-grade inflammation, oxidative imbalance, and progressive metabolic dysfunction [52,53,54]. The connection is biologically relevant because inflammation and oxidative stress are closely intertwined processes that contribute to insulin resistance, mitochondrial impairment, and the progression of metabolic disease [55].

The gut microbiota provides an important link between intestinal disturbances, altered incretin secretion, and systemic metabolic consequences. Studies [56] demonstrated that hydrogen sulfide, produced by *Desulfovibrio*, reduces GLP-1 release and negatively affects host metabolism. Such microbial signalling may therefore interfere directly with incretin availability. Conversely, data showed that tryptophan metabolism promotes GLP-1 secretion and supports β-cell regeneration, suggesting that beneficial microbial pathways may enhance incretin-mediated metabolic adaptation [57]. The gut microbiota–GLP-1 axis can consequently be viewed as a bidirectional regulatory system in which microbial metabolites may either support metabolic homeostasis or contribute to disease development, depending on the metabolic context.

An additional layer of regulation may arise from the relationship between GLP-1 secretion and cellular metabolic homeostasis. Berberine has been investigated as a modulator of metabolic and inflammatory pathways, and evidence suggests that it may regulate oxidative stress and mitochondrial function [38]. These mechanisms may be relevant to maintaining L-cell function and GLP-1 secretory capacity. Meanwhile, GLP-1 production is influenced by signals originating from the intestinal environment. Gut microbiota-derived metabolites, including tryptophan metabolites, have been implicated in regulating the GLP-1 axis [57]. Together, these findings suggest that L-cell function is influenced by both intracellular metabolic status and signals derived from the intestinal environment. The relationship between intestinal microbiota, GLP-1 signalling and metabolic homeostasis further highlights the significance of the intestinal environment in regulating GLP-1-related responses [58]. This is particularly relevant given that L-cells are continuously exposed to fluctuations in nutrient availability, microbial metabolites and inflammatory stimuli. Therefore, changes in the intestinal environment may affect GLP-1 signalling and contribute to metabolic imbalance [57,58].

Further evidence that organelle communication plays a critical regulatory role reinforces the connection between intracellular metabolism and incretin secretion. Endoplasmic reticulum–mitochondria contact sites act as signalling hubs connecting nutrient sensing to GLP-1 secretion in intestinal L-cells [59]. Furthermore, changes to these contact sites have been linked to obesity and type 2 diabetes, indicating that impaired organelle communication could lead to dysregulated GLP-1 secretion in response to metabolic stress [59]. Together, these findings suggest that GLP-1 secretion is not only regulated by extracellular cues, but also by intracellular metabolic processes and organelle homeostasis.

Further in vitro evidence suggests that exposure to λ-carrageenan may influence GLP-1 production and secretion. Exposure to λ-carrageenan significantly reduced proglucagon mRNA expression and GLP-1 secretion in cultured human L-cells (NCI-H716), and similar effects were observed in mouse L-cells (STC-1) [60]. These findings suggest that carrageenan can directly impact L-cell function in an experimental setting. However, as these observations were obtained in cultured cell models, the relevance of these findings to GLP-1 secretion in vivo and the physiological effects of dietary carrageenan in humans remains to be established [60]. Similar effects are observed in response to infectious stressors. In a piglet model, for example, *Salmonella* infection was found to increase blood glucose levels and reduce circulating GLP-1 concentrations while inducing intestinal epithelial damage and pyroptosis of enteroendocrine L-cells [61]. Consistent with these in vivo findings, lipopolysaccharide (LPS) treatment induced pyroptosis and reduced GLP-1 secretion in STC-1 cells in vitro [61]. Thus, infection-associated intestinal inflammation and L-cell injury can impair GLP-1 secretion, potentially contributing to disturbances in glucose homeostasis. Therefore, the available evidence suggests that immune activation, cellular injury and disturbances in redox balance may contribute to impaired incretin output, highlighting the sensitivity of L-cells to nutritional and inflammatory stressors.

Advances in experimental models have substantially improved our understanding of GLP-1 regulation under physiological and pathological conditions. A primary human intestinal epithelial model enriched in L-cells has enabled the detailed characterisation of GLP-1 secretion dynamics, thereby increasing the physiological relevance of in vitro studies [62]. Evidence from microbial research further expands this regulatory landscape by showing that *Akkermansia muciniphila* P9 enhances GLP-1 secretion, providing a beneficial microbial pathway capable of counteracting metabolic inflammation [63]. Environmental factors within the intestinal lumen also directly affect incretin release; for example, reduced extracellular pH inhibits GLP-1 exocytosis [64]. Taken together, these experimental advances demonstrate that GLP-1 biology is highly responsive to changes in intestinal physiology, being shaped by the interplay between diet, microbiota composition, immune activation and cellular stress pathways.

Current evidence suggests that GLP-1 acts as a key coordinator of metabolism, with its activity shaped by an interconnected network of nutritional, hormonal, microbial, inflammatory, and intracellular signals. Activation of the GLP-1R influences glucose homeostasis, appetite regulation, gastrointestinal function, and energy metabolism by coordinating actions in pancreatic, intestinal, hepatic, adipose, and neural tissues [24,40]. Concurrently, GLP-1 signalling is increasingly recognised as a regulator of cellular resilience, influencing mitochondrial function, inflammatory pathways, and oxidative balance [65,66]. The capacity of GLP-1 to integrate metabolic and redox signals may help explain its broad protective effects in obesity, type 2 diabetes, and related metabolic disorders, particularly when its actions are pharmacologically enhanced through receptor agonism.

Notably, the interaction between GLP-1 signalling and oxidative regulation appears to be bidirectional: while GLP-1 can enhance antioxidant defences, improve mitochondrial efficiency, and attenuate inflammatory responses, excessive oxidative stress and metabolic overload can impair GLP-1 secretion and receptor responsiveness. Such reciprocal regulation places GLP-1 within a dynamic feedback system connecting metabolic stress with endocrine adaptation and highlights the importance of redox homeostasis in determining incretin effectiveness [25,65]. Thus, the evidence supports a model in which GLP-1 is not only a mediator of nutrient-dependent endocrine responses but also an important component of the cellular defence network that responds to metabolic and oxidative stress. This perspective provides the basis for the following section, which examines in greater detail how GLP-1 modulates oxidative signalling pathways, mitochondrial homeostasis, and antioxidant responses in metabolic health and disease.

## 4. Oxidative Stress and Metabolic Dysfunction: Pathways and Consequences

### 4.1. Oxidative Stress as a Central Mechanism of Metabolic Dysfunction

Oxidative stress is defined as an imbalance between the production of reactive oxygen species (ROS) and the ability of antioxidant systems to maintain redox homeostasis. There is increasing evidence that redox imbalance is not merely a consequence of metabolic disease, but rather a fundamental mechanism that contributes to its initiation and progression [67]. Importantly, this relationship may extend beyond oxidative stress having a unidirectional effect on metabolic dysfunction. Since redox status influences cellular processes such as nutrient sensing, mitochondrial adaptation, inflammation and endocrine signalling, oxidative stress may affect pathways that regulate metabolic hormones, such as GLP-1. Conversely, alterations in GLP-1 signalling may influence cellular antioxidant capacity, mitochondrial function and inflammatory responses, suggesting a bidirectional relationship between redox homeostasis and metabolic regulation [68]. This reciprocal perspective provides an important framework for understanding how oxidative stress and impaired incretin signalling may interact during metabolic disease progression.

ROS are essential signalling molecules involved in physiological processes such as insulin secretion, mitochondrial adaptation, and immune regulation; however, their excessive accumulation can disrupt cellular signalling, promote molecular and organelle damage, and sustain chronic inflammatory responses [68]. Metabolic overload, nutrient excess, mitochondrial dysfunction and inflammatory activation can reinforce each other, creating a self-perpetuating cycle of ROS production and impaired cellular homeostasis. This is particularly evident in metabolically active organs such as the liver, adipose tissue, pancreas, skeletal muscle and kidney, where it can lead to insulin resistance, impaired energy metabolism and progressive tissue dysfunction [55,68]. These processes are also relevant to enteroendocrine function, as disturbances in the intestinal redox environment can impact the ability of L-cells to respond to nutritional and inflammatory signals.

The close relationship between redox imbalance, inflammation and tissue injury is clearly demonstrated by hepatic metabolic disorders. Authors [3] demonstrated that excessive ROS production activates NF-κB signalling and the NLRP3 inflammasome, thereby promoting pyroptosis and fibrogenic responses in the liver. Comparable mechanisms operate in other metabolically relevant tissues. Oxidative stress contributes to intestinal barrier disruption and chronic inflammation [35], while sustained hyperglycaemia-induced redox imbalance accelerates diabetic kidney injury through mitochondrial damage and inflammatory activation [69]. Thus, evidence from different organ systems supports the view that oxidative stress represents a common molecular link between metabolic disturbances, inflammatory signalling and progressive tissue injury [67,68]. At the same time, these observations provide a broader context for considering how changes in redox homeostasis may influence GLP-1-producing L-cells, and how altered GLP-1 signalling may modulate oxidative and inflammatory pathways in turn.

At the molecular level, the cellular response to redox imbalance is governed by several interconnected regulatory pathways. The most important of these are the NRF2/KEAP1 antioxidant system, the NF-κB inflammatory signalling pathway and the PGC-1α-mediated mitochondrial regulatory pathway [70,71]. Under physiological conditions, the coordinated activity of these pathways helps to maintain antioxidant capacity, mitochondrial function and controlled inflammatory responses. However, when metabolic stress overwhelms these adaptive mechanisms, cellular signalling can shift towards sustained oxidative and inflammatory activation, further compromising metabolic homeostasis and accelerating disease progression. The involvement of these pathways in both cellular redox regulation and metabolic signalling suggests that oxidative stress and GLP-1 dysfunction are interconnected processes rather than independent ones, which sets the scene for examining their bidirectional interactions.

### 4.2. Cellular Sources of ROS in Metabolic Tissues

The generation of ROS in metabolic disorders originates from multiple cellular compartments, reflecting the complexity of metabolic stress responses. Adipocytes, hepatocytes, and skeletal muscle cells are major sources of ROS because of their high metabolic activity and constant exposure to fluctuations in nutrient availability [72,73]. In hepatocytes, mitochondrial electron leakage, NADPH oxidase activation, and inflammatory signalling pathways substantially contribute to the oxidative burden. Excessive ROS accumulation can, in turn, activate inflammasomes and downstream pathways involving caspase-1 and gasdermin-mediated pyroptosis, thereby connecting redox injury with hepatic inflammation and fibrotic responses [3,74]. As the liver plays a central role in glucose and lipid metabolism, disruption of hepatic redox regulation may extend beyond local tissue injury and contribute to systemic metabolic disturbances [72].

Adipose tissue represents another major source of ROS under conditions of metabolic dysfunction. Increased lipid accumulation, inflammatory cytokine production, and activation of the Toll-like receptor 4 (TLR4)/NF-κB signalling pathway enhance ROS generation and promote chronic, low-grade inflammation. Activation of related inflammatory pathways has also been implicated in hyperuricaemic nephropathy, in which TLR4/NF-κB signalling contributes to oxidative injury and tissue damage [75]. Skeletal muscle, the principal site of insulin-mediated glucose disposal, is particularly susceptible to redox disturbances. Mitochondrial dysfunction, impaired antioxidant enzyme activity, and inflammatory signalling can compromise metabolic flexibility and reduce the efficiency of glucose utilisation [76]. Environmental stressors may further exacerbate redox imbalance in muscle and other metabolically active tissues. For instance, exposure to particulate matter increases malondialdehyde levels and decreases SOD2 activity, thereby amplifying intracellular oxidative stress [77]. Thus, evidence from the liver, adipose tissue, skeletal muscle, and kidney indicates that ROS generation in metabolic disease is not confined to a single cellular or organ source. Rather, it reflects interconnected disturbances across multiple metabolically active tissues, with local oxidative injury potentially reinforcing systemic metabolic dysfunction. The resulting redox environment provides an important basis for understanding insulin resistance and the metabolic complications associated with obesity and type 2 diabetes.

### 4.3. Mitochondrial Dysfunction and Impaired Redox Homeostasis

As major sites of ROS production, mitochondria are particularly vulnerable to metabolic overload, making mitochondrial dysfunction an important link between redox imbalance and cellular stress [78,79]. Impaired mitochondrial quality control, including disturbances in mitophagy and redox-linked signalling, can further compromise organelle integrity [80]. Experimental studies support the importance of protecting mitochondria and triggering antioxidant responses in order to limit oxidative and inflammatory injury. Treatment with essential oils has been reported to reduce mitochondrial injury and pyroptosis in inflammatory settings [81], while SIRT1/p62-dependent mitophagy induced by resveratrol protects hepatocytes from LPS-related oxidative damage [82]. Similarly, PPARγ activation reduces ROS and inflammatory mediator expression [83], whereas Mo3Se4 nanoparticles improve redox balance through Nrf2-Keap1 activation [84]. These findings emphasise the close interconnection between mitochondrial integrity and redox control as determinants of cellular metabolic adaptation. This relationship is particularly relevant to GLP-1 biology, given that GLP-1 signalling has also been associated with the regulation of mitochondrial function, antioxidant responses and cellular stress resistance.

### 4.4. Nrf2-Keap1/NF-κB/NLRP3

The Nrf2-Keap1, NF-κB and NLRP3 pathways are important links between redox imbalance and inflammatory activation [69,85]. Although Nrf2 promotes antioxidant defence by inducing genes such as HO-1, NQO1, GCLc, and GCLm, persistent metabolic stress can overwhelm this adaptive response [86,87]. Evidence from experimental models indicates that activation of Nrf2-dependent signalling reduces inflammatory and oxidative injury. This has been observed with scopoletin [88], linarin [89], and hyperoside [90]. Conversely, activation of TLR4/NF-κB increases the expression of inflammatory mediators such as IL-1β, IL-6, and TNF-α, thereby exacerbating oxidative stress [91,92]. NLRP3 responds to excessive ROS, mitochondrial injury, and impaired autophagy, leading to caspase-1 activation and GSDMD-dependent pyroptosis [3,93]. The convergence of these pathways therefore provides a basis for linking metabolic stress, mitochondrial dysfunction, oxidative imbalance, and inflammation. Importantly, these processes also intersect with pathways influenced by GLP-1 signalling, providing a basis for considering redox and inflammatory regulation as part of a reciprocal relationship between oxidative stress and incretin biology.

### 4.5. Oxidative Stress, Insulin Resistance and Type 2 Diabetes

Oxidative stress contributes to insulin resistance and type 2 diabetes by disrupting insulin signalling and increasing inflammatory activation, as well as impairing mitochondrial energy metabolism [94,95]. Hyperglycaemia can further increase mitochondrial ROS and NF-κB activity, whilst simultaneously weakening Nrf2-dependent antioxidant responses [94,95,96]. Furthermore, inflammatory cytokines, including IL-1β, IL-6 and TNF-α, can interfere with IRS signalling and impair glucose and lipid metabolism [97,98]. Skeletal muscle is particularly relevant due to its significant involvement in insulin-stimulated glucose disposal; mitochondrial dysfunction can reduce ATP production, glucose oxidation and insulin-stimulated glucose uptake [10,11,99]. Environmental stressors such as particulate matter can increase the oxidative burden by suppressing Nrf2 signalling [100,101], whereas restoring Nrf2/Keap1 activity has been linked to improved metabolic parameters and reduced inflammation [102]. These findings support the idea of a reinforcing relationship in which oxidative stress, mitochondrial dysfunction, inflammation and impaired insulin signalling can sustain metabolic dysfunction.

The interaction between these pathways and GLP-1 signalling provides a broader framework for understanding this process. Mitochondrial ROS formation, Nrf2-linked antioxidant defence, NF-κB-mediated inflammation and NLRP3 activation link metabolic overload to cellular dysfunction [103]. Conversely, GLP-1 receptor activation has been linked to enhanced mitochondrial function, increased antioxidant capacity, reduced inflammatory signalling and greater metabolic flexibility [104]. Through these effects, GLP-1 may contribute to maintaining redox homeostasis in metabolically stressed tissues, which is relevant to obesity, insulin resistance, and type 2 diabetes [25]. Therefore, the relationship between oxidative stress and GLP-1 signalling could extend beyond the impact of redox imbalance on incretin secretion. GLP-1 signalling may also influence the cellular pathways that determine oxidative and mitochondrial stress. This reciprocal interaction suggests that redox imbalance and GLP-1 dysregulation are interconnected processes in metabolic disease.

## 5. Bidirectional Interactions Between GLP-1 Signalling and Oxidative Pathways

### 5.1. GLP-1 as a Regulator of Mitochondrial Redox Homeostasis

GLP-1 has functions that extend beyond its traditional role as an incretin, influencing mitochondrial function, oxidative signalling and inflammatory responses in metabolically active tissues [105]. In type 2 diabetes, chronic hyperglycaemia increases mitochondrial ROS production and impairs bioenergetic function [106]. Activation of the GLP-1 receptor (GLP-1R) has been associated with improved mitochondrial efficiency, reduced ROS generation and enhanced antioxidant responses. These effects were first demonstrated in vascular cells through modulation of LOX-1 signalling [107]. These responses involve the regulation of mitochondrial respiration and the activation of the cAMP-PKA-CREB pathway, which supports mitochondrial adaptation and antioxidant defences in skeletal muscle, the liver and cardiovascular tissues [65,105,108].

Rather than acting as a direct ROS scavenger [109], GLP-1 appears to preserve mitochondrial stability and limit oxidative injury [110]. Experimental studies in neuronal and cardiac models demonstrate improved mitochondrial integrity and membrane potential preservation, reduced ROS accumulation and protection against inflammatory injury following GLP-1R activation [111,112,113]. These responses overlap with Nrf2-dependent antioxidant defence, AMPK signalling, and sirtuin-regulated mitochondrial control, thereby linking GLP-1 signalling to pathways that maintain redox homeostasis [25,65,105].

GLP-1 also supports mitochondrial quality control by regulating autophagy, mitophagy and biogenesis [66,114,115]. Activation of the GLP-1 receptor (GLP-1R) promotes p62- and LC3-dependent autophagic flux, modulates AMP-activated protein kinase (AMPK)-associated stress responses and lysosomal function, and stimulates mitochondrial biogenesis through peroxisome proliferator-activated receptor gamma coactivator 1-alpha (PGC-1α), nuclear respiratory factor 1 (NRF1), and transcription factor A (TFAM) [105,116,117,118,119]. Studies using various GLP-1R agonists, such as exendin-4 and liraglutide, have demonstrated improved mitochondrial function, restored membrane potential and reduced oxidative injury in experimental disease models. However, these effects have been observed using different pharmacological compounds and experimental settings. These findings support the idea that mitochondrial quality control is an important area of interaction between GLP-1 signalling and redox regulation.

### 5.2. Organ-Specific Protection Mediated by GLP-1 Signalling

Although the supporting evidence comes from different GLP-1 receptor agonists and experimental models, the effects of GLP-1 signalling on mitochondria and redox balance have been reported across multiple organ systems [65,105]. Despite differences between tissues, consistent findings include the preservation of mitochondrial integrity, the reduction in oxidative stress and the attenuation of inflammatory injury [120]. In nervous tissue, GLP-1R agonists have been associated with reduced ferroptosis, improved mitochondrial dynamics and reduced oxidative injury in models of ischaemic and diabetic neurological damage [121,122,123]. In cardiovascular tissues, treatment with various GLP-1R agonists has been linked to the preservation of mitochondrial membrane potential, improved endothelial nitric oxide bioavailability, enhanced antioxidant responses and reduced inflammatory signalling. Studies have reported benefits in hypoxia/reoxygenation and ischaemia/reperfusion models [112,115,117,124,125,126].

Similar patterns have been observed in renal tissues, where exenatide and polyethylene glycol-exenatide have been shown to enhance mitochondrial function, nitric oxide production, and redox balance in the presence of diabetes or lipotoxicity [127,128,129]. Further evidence from reproductive and endocrine models suggests improved mitochondrial function in Leydig cells and enhanced steroidogenic regulation via FDX1 in experimental polycystic ovary syndrome [130,131]. While these observations are derived from various pharmacological agents and tissue-specific models, they consistently suggest that GLP-1 signalling interacts with mitochondrial function and oxidative regulation beyond its recognised role in glucose-dependent insulin secretion [105].

Table 1 summarises the evidence linking GLP-1 signalling to oxidative stress, mitochondrial homeostasis and inflammatory pathways in experimental and clinical models. These findings suggest that GLP-1R activation plays a part in preserving mitochondrial function, limiting oxidative damage and maintaining metabolic homeostasis.

### 5.3. Gut Microbiota, Nutrition and GLP-1-Dependent Redox Regulation

GLP-1 signalling is involved in the gut–microbiota–metabolism axis, in which microbial metabolites influence the secretion of endogenous GLP-1 and contribute to systemic redox regulation [141,142,143]. Short-chain fatty acids stimulate GLP-1 release and support mitochondrial function, thereby limiting ROS formation and improving metabolic flexibility [144]. However, dysbiosis can disrupt these interactions by altering AMPK signalling, thereby impairing mitochondrial function and enhancing neuroinflammation [145]. Changes in gut permeability and NLRP3 activation may further modify GLP-1 responsiveness and systemic oxidative status [141,146]. Dietary factors such as omega-3 fatty acids, flavonoids, anthocyanins, probiotics and high-quality protein can reinforce GLP-1-mediated metabolic adaptations by affecting mitochondrial biogenesis, suppressing cytokines and maintaining lean mass [141,142,147]. These nutritional influences may complement GLP-1RAs therapy, broadening improvements in redox and metabolic stability.

**Figure 1:**
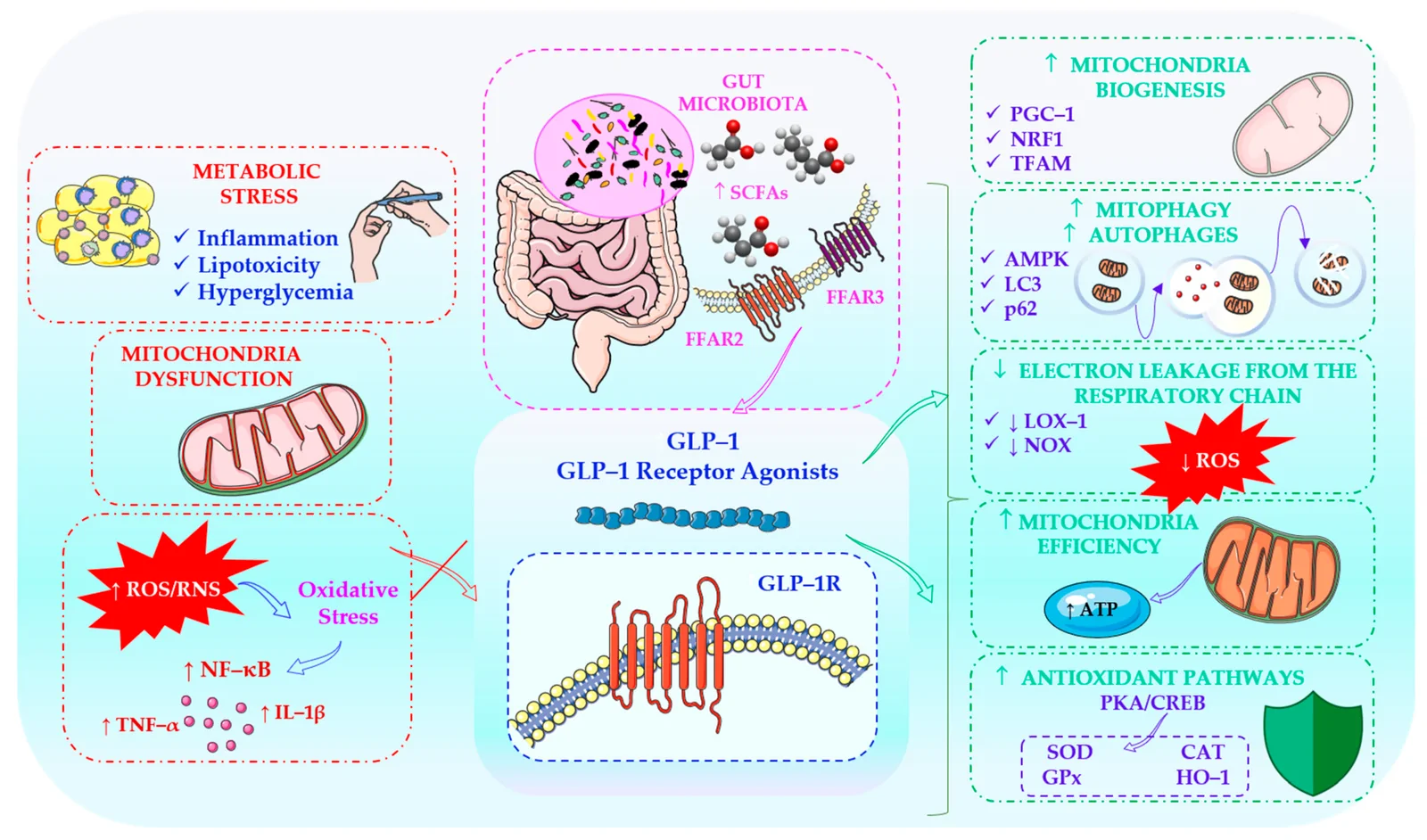
Schematic depicting the mechanisms by which GLP-1 signalling and GLP-1 receptor agonists (GLP-1RAs) mitigate oxidative stress. Activation of the GLP-1 receptor improves mitochondrial function and homeostasis, enhances biogenesis and mitophagy, reduces reactive oxygen species (ROS) production, and enhances antioxidant mechanisms, thereby protecting cells and organs from metabolic stress-induced damage. Image provided by Servier Medical Art (https://smart.servier.com/), licenced under CC BY 4.0 (https://creativecommons.org/licences/by/4.0/, accessed on 31 July 2026). Abbreviations: AMPK–AMP-activated protein kinase; ATP–adenosine triphosphate; CAT–catalase; CREB–cAMP response element-binding protein; FFAR2–free fatty acid receptor 2; FFAR3–free fatty acid receptor 3; GLP-1–glucagon-like peptide-1; GLP-1R–glucagon-like peptide-1 receptor; GPx–glutathione peroxidase; HO-1–heme oxygenase-1; IL-1β–interleukin-1 beta; LC3–microtubule-associated protein 1 light chain 3; LOX-1–lectin-like oxidised low-density lipoprotein receptor-1; NF-κB–nuclear factor kappa-light-chain-enhancer of activated B cells; NOX–nicotinamide adenine dinucleotide phosphate (NADPH) oxidase; NRF1–nuclear respiratory factor 1; p62 (SQSTM1)–sequestosome 1; PGC-1α–peroxisome proliferator-activated receptor gamma coactivator-1 alpha; PKA–protein kinase A; RNS–reactive nitrogen species; ROS–reactive oxygen species; SCFAs–short-chain fatty acids; SOD–superoxide dismutase; TFAM–mitochondrial transcription factor A; TNF-α–tumour necrosis factor alpha.

Evidence indicates that GLP-1 regulates oxidative homeostasis through its coordinated effects on mitochondrial stability, stress-response pathways and inflammation (Figure 1). GLP-1RAs inhibit ferroptosis via β-Klotho–AMPK signalling, modulate autophagy and enhance nitric oxide/cGMP-dependent endothelial function [148]. Additional vascular redox effects have been reported in studies by Cai et al. (2018) [149] and Battistoni et al. (2025) [150]. These molecular responses correspond with improvements in clinical biomarkers, including reductions in ALT and AST, increased albuminuria and enhanced flow-mediated dilation, as well as decreased markers of microglial activation such as Iba1 and CD68. Overall, experimental and translational findings support the involvement of oxidative signalling in the therapeutic actions of GLP-1RAs in various organs [151].

GLP-1 influences several cellular processes that promote mitochondrial stability, inhibit ROS formation, boost endogenous antioxidant activity, regulate autophagy and minimise chronic inflammatory signalling. The effectiveness of these actions has been well documented in the context of obesity, type 2 diabetes, cardiovascular disease, metabolic dysfunction-associated steatotic liver disease and neurodegenerative conditions [152,153]. There is therefore growing interest in combining GLP-1RAs with interventions that influence gut microbiota, nutrition-derived metabolic cues and redox-related pathways, as such approaches may affect mitochondrial performance, antioxidant capacity and inflammatory tone simultaneously. Evidence also suggests that GLP-1 signalling contributes to preserving mitochondrial integrity, limiting ROS accumulation and supporting cellular resilience under sustained metabolic stress. These interactions span multiple organ systems and help to contextualise the broad physiological reach of GLP-1-related pathways [154,155,156].

## 6. Activation of the Nrf2–Keap1 Antioxidant Axis by GLP-1

### 6.1. Activation of the Nrf2–Keap1 Antioxidant Pathway

GLP-1RAs are important modulators of the Nrf2–Keap1 antioxidant signalling axis. Through this interaction, incretin signalling is linked to endogenous cellular defence mechanisms that protect against oxidative stress [65]. In addition to their well-established glucose-lowering effects, GLP-1RAs promote the nuclear translocation and transcriptional activity of Nrf2, thereby enhancing the expression of antioxidant and cytoprotective genes, improving mitochondrial function, and limiting ROS accumulation. The resulting response strengthens cellular redox homeostasis and provides a mechanistic connection between mitochondrial preservation and endogenous antioxidant defence. Activation of Nrf2 is closely coordinated with modulation of its cytoplasmic inhibitor, Kelch-like ECH-associated protein 1 (Keap1), which acts as a principal sensor of intracellular oxidative stress [65,105]. Under physiological conditions, Keap1 targets Nrf2 for ubiquitination and proteasomal degradation. By preserving mitochondrial function and limiting ROS-dependent disruption of Keap1 regulation, GLP-1RAs may favour sustained Nrf2 activity. Prolonged Nrf2 signalling promotes the expression of antioxidant enzymes involved in glutathione metabolism, detoxification, and mitochondrial protection [37,69,105]. Enhanced antioxidant capacity may consequently reduce oxidative injury and increase the resilience of metabolically active tissues exposed to chronic metabolic stress.

### 6.2. Regulation of Antioxidant Defence and Mitochondrial Protection

The functional significance of Nrf2 activation is particularly evident in its ability to support mitochondrial integrity across different experimental models. In cardiovascular tissues, GLP-1 signalling reduces ROS generation by improving mitochondrial homeostasis and respiratory efficiency [65,105]. In renal tubular cells, exenatide activates SIRT1, thereby promoting Nrf2-dependent antioxidant responses and protecting mitochondria against oxidative damage induced by lipotoxicity [127]. Lixisenatide has likewise been reported to enhance SIRT3/FoxO3 signalling in endothelial progenitor cells, reducing mitochondrial dysfunction and improving antioxidant capacity [129]. These observations indicate that GLP-1R activation can engage Nrf2-dependent antioxidant responses through several upstream regulators, including SIRT1 and SIRT3. The resulting improvement in mitochondrial integrity, reduction in ROS accumulation, and greater cellular resistance to oxidative injury suggest that GLP-1 coordinates multiple components of the antioxidant response rather than acting through a single molecular target [65,105].

Importantly, the antioxidant effects mediated by Nrf2 extend beyond the direct neutralisation of ROS. Activation of Nrf2 promotes the transcription of numerous cytoprotective genes that encode detoxifying and antioxidant enzymes. Enhanced expression of these enzymes helps restore intracellular redox balance and limit oxidative damage to proteins, lipids, and mitochondrial DNA [157]. GLP-1RAs may consequently create a cellular environment that favours metabolic adaptation and the long-term maintenance of mitochondrial function under chronic metabolic stress. Furthermore, an increasing body of evidence suggests that the antioxidant properties of GLP-1 are closely associated with mitochondrial quality-control mechanisms. By reducing mitochondrial ROS production and preserving mitochondrial membrane potential, activation of the GLP-1 receptor interrupts the vicious cycle in which oxidative stress further impairs mitochondrial function, leading to progressive ROS amplification [113,117]. Such feed-forward amplification represents an important biological mechanism contributing to metabolic deterioration in obesity, type 2 diabetes, and associated cardiovascular complications.

Thus, the activation of the Nrf2–Keap1 pathway appears to represent an important mechanism through which GLP-1RAs support mitochondrial homeostasis and redox balance. Simultaneous enhancement of endogenous antioxidant defences and mitochondrial function may create a more favourable intracellular environment, helping to protect metabolically active tissues from persistent oxidative damage and providing a mechanistic basis for the broader cytoprotective effects of GLP-1 signalling [117,158].

### 6.3. GLP-1-Mediated Suppression of NF- κB Signalling

Because oxidative stress and inflammation are closely interconnected, the effects of GLP-1 on Nrf2–Keap1 signalling extend to the regulation of major pro-inflammatory pathways, particularly nuclear factor kappa-light-chain-enhancer of activated B cells (NF-κB). Reciprocal regulation between antioxidant and inflammatory signalling represents an important mechanism through which GLP-1RAs may limit oxidative injury and attenuate chronic inflammation [159]. Alongside Nrf2 activation, GLP-1 signalling suppresses NF-κB activity, thereby reducing the transcription of pro-inflammatory cytokines and limiting ROS-driven inflammatory amplification [160]. By limiting mitochondrial ROS generation and improving mitochondrial function, GLP-1RAs may also reduce an important stimulus for persistent NF-κB activation, shifting the intracellular balance towards antioxidant defence rather than chronic inflammatory signalling [117,132].

Experimental evidence supports the interaction between GLP-1 signalling, mitochondrial protection, and NF-κB regulation. For example, in cardiomyocytes exposed to interleukin-1β (IL-1β), liraglutide prevented metabolic disturbances and preserved mitochondrial function by suppressing NF-κB signalling [112,161]. Similarly, exendin-4 reduced cisplatin-induced cardiac injury by limiting ROS accumulation, preventing apoptosis, and attenuating NF-κB activation [120]. In retinal ganglion cells, liraglutide inhibited NF-κB-associated autophagy and reduced oxidative damage under diabetic conditions [139]. Thus, the evidence indicates that GLP-1 signalling may influence both sides of the redox–inflammatory interface: strengthening endogenous antioxidant defences while simultaneously limiting inflammatory pathways that can sustain ROS production, mitochondrial dysfunction, and tissue injury.

### 6.4. Modulation of the NLRP3 Inflammasome and Mitochondrial–Inflammatory Crosstalk

Suppressing NF-κB is important because NF-κB-dependent priming is necessary for the activation of NOD-, LRR- and pyrin domain-containing protein 3 (NLRP3) inflammasomes. Accordingly, GLP-1 receptor activation may influence both the initiation and amplification of inflammasome signalling, further positioning GLP-1 as an important regulator of inflammation-associated ROS production [155]. GLP-1 receptor activation reduces mitochondrial dysfunction and limits the release of mitochondrial danger-associated molecular patterns (DAMPs), which would otherwise trigger NLRP3 inflammasome assembly. By simultaneously reducing mitochondrial ROS production and attenuating NF-κB-dependent priming, GLP-1RAs weaken the molecular conditions required for efficient inflammasome activation. Consequently, activation of caspase-1 and the maturation of interleukin-1β (IL-1β) and interleukin-18 (IL-18) are reduced. The convergence of mitochondrial and inflammatory signalling places GLP-1 within a regulatory network that directly connects mitochondrial integrity with inflammasome activity [113,161].

Evidence from diverse disease models further supports the interaction between mitochondrial protection and inflammasome regulation. In an experimental model of ischaemic brain injury, semaglutide reduced ferroptosis by modulating Forkhead Box Protein O1 (FoxO1) and Dynamin-Related Protein 1 (DRP1), thereby preserving mitochondrial integrity and limiting ROS-driven inflammasome activation [117,125]. GLP-1 signalling has also been shown to reduce mitochondrial stress and inflammasome-associated autophagy in pulmonary arterial hypertension through regulation of Atg5, Atg7, Beclin1 and LC3β [114]. In cases of diabetic cognitive impairment, liraglutide has been shown to reduce lipid peroxidation and ROS generation, thereby indirectly suppressing NLRP3 inflammasome activation and limiting neuroinflammatory injury [123]. Thus, the available evidence suggests that NLRP3 inhibition may result primarily from improved mitochondrial homeostasis rather than representing an isolated pharmacological effect. Preservation of mitochondrial membrane potential, reduction in ROS accumulation, and attenuation of inflammatory signalling can jointly disrupt the self-perpetuating cycle in which mitochondrial dysfunction amplifies oxidative stress and chronic inflammation [162].

The close relationship between mitochondrial function and inflammatory signalling is further supported by studies demonstrating bidirectional communication between the two systems. Activation of the GLP-1R improves mitochondrial performance, limits ROS-dependent activation of NF-κB, and reduces the release of mitochondrial danger signals that would normally reinforce inflammatory cascades [25,69]. Stabilisation of the mitochondrial membrane potential and reduction in oxidative stress therefore weaken the positive feedback loop connecting mitochondrial injury with inflammasome activation, ultimately limiting progressive tissue damage [163].

Experimental observations obtained from neuronal, cardiovascular and endocrine models provide further support for this integrated mechanism. In neuroblastoma cells exposed to high glucose, exendin-4 restored the mitochondrial membrane potential and reduced ROS accumulation by activating the exchange protein directly activated by cAMP (Epac)/Akt pathway [122]. Similarly, exenatide improved mitochondrial function and reduced apoptosis in cardiomyocytes subjected to hypoxia/reoxygenation injury [124], while liraglutide enhanced mitochondrial performance and reduced oxidative stress in metabolic dysfunction-affected Leydig cells [130]. Although the experimental settings and affected tissues differ, the studies point to a common biological response in which preservation of mitochondrial integrity is associated with reduced NF-κB activation, attenuation of NLRP3 inflammasome signalling, and restoration of redox homeostasis.

Thus, the available evidence indicates that GLP-1RAs coordinate antioxidant and anti-inflammatory responses through an interconnected signalling network involving activation of the Nrf2–Keap1 pathway, suppression of NF-κB, and inhibition of NLRP3 inflammasome activity. Rather than acting on isolated molecular targets, GLP-1 orchestrates a comprehensive adaptive response that restores mitochondrial function, limits the generation of ROS, and interrupts the feed-forward cycle linking oxidative stress with chronic inflammation [25,65,164]. Such coordinated regulation provides a plausible biological basis for the broad therapeutic effects of GLP-1RAs across metabolic, cardiovascular, renal and neurodegenerative disorders.

### 6.5. Systemic Regulation of Redox–Inflammatory Homeostasis

GLP-1RAs act as systemic regulators of mitochondrial behaviour, redox balance and inflammatory tone in cardiovascular, renal, hepatic, neural and endocrine models. Activation of the GLP-1 receptor improves mitochondrial efficiency, limits the formation of excessive ROS and attenuates redox-sensitive inflammatory pathways [117,165]. These effects are not limited to direct antioxidant activity; rather, they reflect a coordinated response that enhances mitochondrial integrity, restores intracellular redox balance, and reduces inflammatory amplification. Organs with high metabolic demands, including the heart, liver, kidneys, brain and vascular endothelium, may therefore exhibit increased resilience to oxidative injury and chronic inflammation [25,65].

Conserved intracellular pathways further illustrate how GLP-1 shapes mitochondrial and redox regulation across diverse disease models. In vascular smooth muscle cells, for example, GLP-1 signalling interacts with LOX-1 to reduce mitochondrial ROS generation [107]. In Alzheimer’s disease models, activation of the cAMP–PKA pathway improves astrocytic mitochondrial function and supports neuronal metabolism [111], whereas dual GLP-1/GIP agonists reduce mitochondrial stress via Akt/JNK signalling in Parkinson’s disease models [134]. These actions converge on the preservation of mitochondrial function, the attenuation of oxidative stress, and the suppression of inflammatory signalling. This forms a cytoprotective network that includes the activation of Nrf2–Keap1, the inhibition of NF-κB, and the limitation of NLRP3 inflammasome assembly [25,65,117]. By regulating oxidative signalling, mitochondrial homeostasis and inflammatory responses in this integrated way, GLP-1-based therapies could benefit conditions characterised by excessive oxidative stress and chronic low-grade inflammation [166,167].

## 7. GLP-1RAs and Redox-Related Effects

### 7.1. GLP-1RAs as Modulators of Oxidative Homeostasis

GLP-1RAs, including liraglutide, semaglutide, dulaglutide and the dual GIP/GLP-1 receptor agonist tirzepatide, have become key pharmacological modulators of oxidative homeostasis in obesity, type 2 diabetes and associated cardiometabolic disorders [117,119,165]. While these agents were initially developed to improve glycaemic control, increasing evidence indicates that their therapeutic actions extend well beyond glucose regulation. By improving mitochondrial efficiency, limiting electron leakage from the respiratory chain, and enhancing endogenous antioxidant defence systems, GLP-1RAs consistently shift the intracellular redox balance towards a less oxidative state [105,168]. The broader biological activity of these therapies reflects the close interplay between mitochondrial function, oxidative stress and chronic metabolic inflammation described in the preceding sections. Persistent hyperglycaemia, excessive nutrient availability and insulin resistance promote continuous ROS production, mitochondrial dysfunction and activation of redox-sensitive inflammatory pathways. Activation of the GLP-1 receptor interrupts these pathological processes by restoring mitochondrial homeostasis, reducing oxidative injury and attenuating inflammatory signalling, thereby improving cellular resilience across multiple metabolically active tissues [169,170,171].

Evidence from experimental and clinical studies shows that GLP-1RAs reduce lipid peroxidation and oxidative damage, while increasing antioxidant capacity. Consistent reductions in circulating malondialdehyde (MDA), oxidised low-density lipoprotein (oxLDL) and 8-isoprostaglandin F2α (8-iso-PGF2α) have been reported following GLP-1RA therapy. Such biochemical changes are closely associated with better metabolic control, reduced systemic inflammation and improved endothelial function [172,173]. Taken together, the findings support the view that GLP-1RAs function not only as incretin-based glucose-lowering agents, but also as systemic modulators of redox homeostasis that integrate mitochondrial protection, antioxidant signalling and inflammatory regulation [171,174]. This interpretation is further supported by recent evidence syntheses. Himmerich (2025) emphasised that GLP-1RAs consistently reduce oxidative stress, apoptosis and inflammatory activation, thereby contributing to metabolic and neuroprotective effects [175]. Similarly, systematic review, which encompassed 62 primary studies, demonstrated that dulaglutide, exenatide, liraglutide and semaglutide enhance antioxidant enzyme activity while reducing lipid peroxidation and protein oxidation [176]. Thus, the available evidence identifies modulation of oxidative stress as an important mechanism contributing to the diverse biological effects of GLP-1 receptor agonists.

### 7.2. Comparative Redox Effects of Individual GLP-1 Receptor Agonists

All currently available GLP-1RAs improve oxidative homeostasis, but their redox-related effects differ in terms of magnitude, duration, and underlying pharmacological characteristics. Such differences may reflect variations in receptor affinity, pharmacokinetic profiles, molecular structure and treatment duration rather than fundamentally distinct mechanisms of action [177,178]. Of the agents currently available, semaglutide has demonstrated particularly pronounced improvements in metabolic and inflammatory outcomes, which may contribute to greater reductions in oxidative burden compared with earlier GLP-1RAs [177]. Comparative analyses indicate that semaglutide produces stronger decreases in inflammatory mediators and metabolic risk factors than liraglutide or dulaglutide. The associated improvements in oxidative biomarkers may be related, at least in part, to semaglutide’s longer biological half-life, which provides sustained GLP-1 receptor stimulation and prolonged metabolic effects [178]. Likewise, liraglutide exerts significant antioxidant effects, as evidenced by reductions in MDA concentrations and improvements in glutathione-dependent antioxidant capacity. However, its shorter half-life may limit the persistence of such responses compared with once-weekly formulations [177]. Dulaglutide also improves oxidative balance and metabolic control; however, available evidence suggests that its effects on circulating oxidative biomarkers are generally more moderate than those reported for semaglutide [179,180]. Nevertheless, all three agents consistently reduce oxidative stress through mechanisms involving improved mitochondrial function, attenuation of chronic inflammation, and restoration of redox homeostasis [177,178].

**Figure 2:**
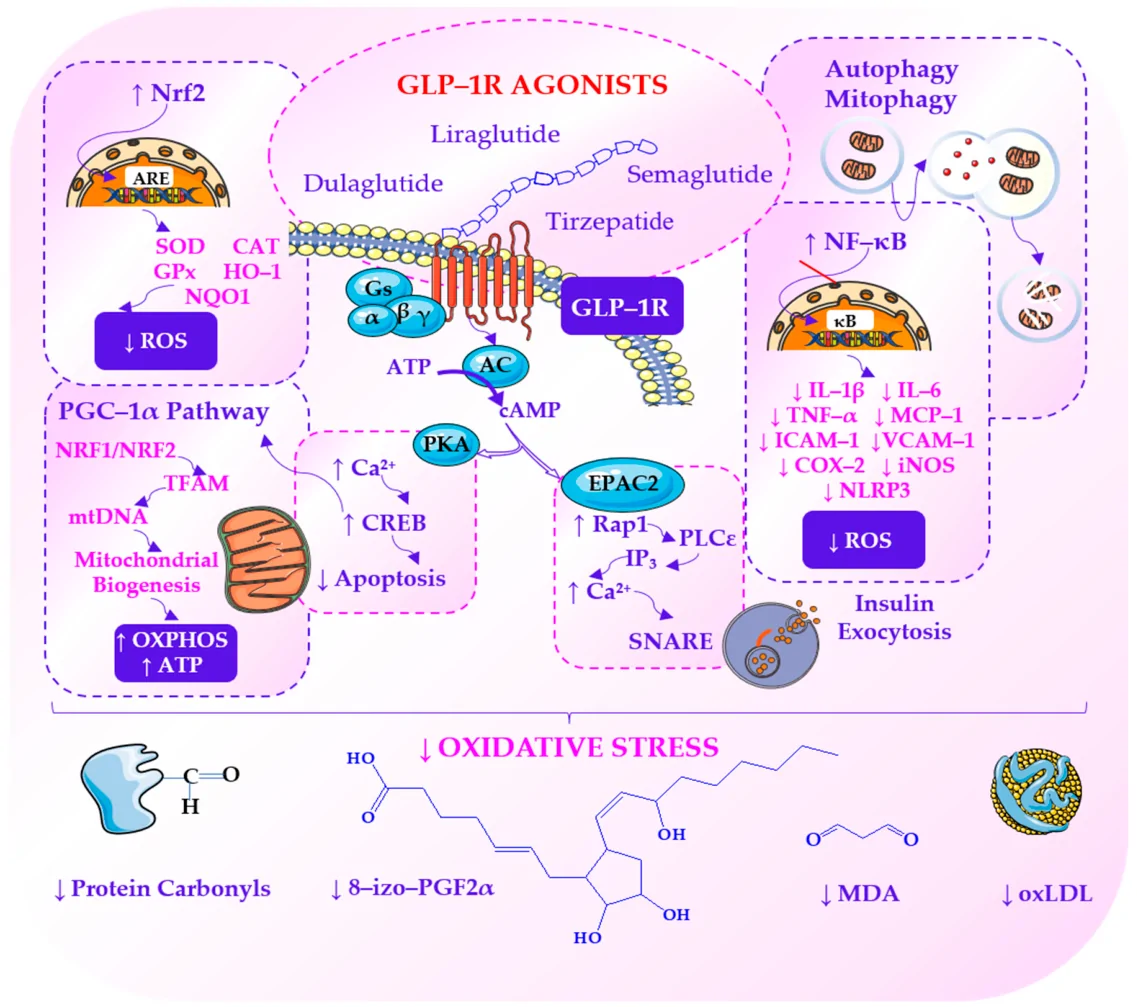
Mechanism of action of GLP-1 receptor agonists (GLP-1RAs) leading to improved mitochondrial function, reduced oxidative stress, and inflammation. GLP-1 receptor agonists (GLP-1RAs) bind to the GLP-1R receptor, activating Gs protein, adenylate cyclase, and the cAMP-dependent signalling pathway. Subsequent activation of PKA and EPAC2 leads to increased insulin secretion and initiates mechanisms that improve mitochondrial function, activate the antioxidant response, and inhibit proinflammatory pathways. Consequently, the production of reactive oxygen species (ROS) is reduced, lipid peroxidation is reduced, and the concentration of oxidative stress markers (MDA, oxLDL, and 8-iso-PGF2α) is decreased, which translates into improved endothelial function, redox homeostasis, and metabolic control. Image provided by Servier Medical Art (https://smart.servier.com/), licenced under CC BY 4.0 (https://creativecommons.org/licences/by/4.0/, accessed on 31 July 2026). Abbreviations: 8-iso-PGF2α–8-isoprostaglandin F2 alpha; AC–adenylyl cyclase; ARE–antioxidant response element; ATP–adenosine triphosphate; cAMP–cyclic adenosine monophosphate; CAT–catalase; COX-2–cyclooxygenase-2; CREB–cAMP response element-binding protein; EPAC2–exchange protein directly activated by cAMP 2; GLP-1R–glucagon-like peptide-1 receptor; GPx–glutathione peroxidase; Gs–stimulatory G protein (Gαs); HO-1–heme oxygenase-1; ICAM-1–intercellular adhesion molecule-1; IL-1β–interleukin-1 beta; IL-6–interleukin-6; iNOS–inducible nitric oxide synthase; IP_3_–inositol 1,4,5-trisphosphate; MCP-1–monocyte chemoattractant protein-1 (CCL2); MDA–malondialdehyde; mtDNA–mitochondrial DNA; NF-κB–nuclear factor kappa B; NLRP3–NOD-, LRR- and pyrin domain-containing protein 3; NQO1–NAD(P)H quinone dehydrogenase 1; NRF1–nuclear respiratory factor 1; Nrf2–nuclear factor erythroid 2–related factor 2; ox-LDL–oxidised low-density lipoprotein; OXPHOS–oxidative phosphorylation; PGC-1α–peroxisome proliferator-activated receptor gamma coactivator-1 alpha; PKA–protein kinase A; PLCε–Phospholipase C; Rap1–Ras-related protein 1; ROS–reactive oxygen species; SNARE–soluble N-ethylmaleimide-sensitive factor attachment protein receptor; SOD–superoxide dismutase; TFAM–mitochondrial transcription factor A; TNF-α–tumour necrosis factor alpha; VCAM-1–vascular cell adhesion molecule-1.

Figure 2 illustrates the mechanism of action of GLP-1 receptor agonists, highlighting the GLP-1R/Gs/cAMP/PKA and EPAC2 signalling pathways that contribute to improved mitochondrial function, enhanced antioxidant defence, and reduced oxidative stress and inflammation, ultimately supporting redox homeostasis, endothelial function, and metabolic control.

The therapeutic landscape has expanded with the introduction of tirzepatide, a dual GIP/GLP-1 receptor agonist which activates two incretin pathways simultaneously, thereby extending the biological actions of conventional GLP-1RAs [181]. Dual incretin signalling enhances glucose-dependent insulin secretion, suppresses glucagon release and improves mitochondrial efficiency [176], indicating a greater ability to modulate redox-sensitive pathways than single-agonist therapies have. Furthermore, experimental evidence indicates that tirzepatide has stronger anti-inflammatory effects than semaglutide in diabetes models [171], supporting the idea that activating both the GIP and GLP-1R may offer greater protection against metabolic and inflammatory stress. Clinical findings support these mechanistic observations. In the SURMOUNT-1 trial, for example, tirzepatide produced substantial and sustained reductions in body weight alongside marked improvements in glycaemic control [174]. As obesity, insulin resistance and chronic hyperglycaemia are major drivers of oxidative stress, these metabolic improvements likely contribute to secondary reductions in oxidative burden and inflammatory activation. Thus, the available evidence suggests that tirzepatide is one of the most promising dual-incretin therapies for improving metabolic regulation and potentially redox homeostasis in obesity and type 2 diabetes [181].

A growing body of literature indicates that discontinuation of GLP-1RAs therapy is frequently driven by adverse effects, as documented in recent clinical and pharmacovigilance analyses and summarised in a review [182]. Reported data consistently show that gastrointestinal intolerance—most notably nausea, vomiting, diarrhoea and constipation—constitutes the predominant reason for treatment cessation and displays a clear dose-dependent pattern. Several less common but clinically relevant complications have also been described, including biliary and gallbladder events, acute pancreatitis, intestinal obstruction, acute kidney injury, transient worsening of diabetic retinopathy, and thyroid C-cell alterations. Newer safety signals, such as perioperative aspiration, neuropsychiatric manifestations and mild tachycardia, have also emerged in recent reports [182]. These findings therefore demonstrate that interruption of GLP-1RA or tirzepatide therapy reflects a substantial, multifactorial burden of adverse effects influenced by the specific agent, titration strategy, comorbid conditions, and off-label use.

Despite these quantitative differences, the available evidence suggests that all GLP-1RAs operate via a broadly similar biological framework. Regardless of their pharmacological profile, they improve mitochondrial efficiency, reduce excessive ROS production, enhance endogenous antioxidant defences, and attenuate redox-sensitive inflammatory signalling [168]. Therefore, the principal distinction among individual agents lies in the magnitude and durability of their effects rather than in fundamentally different mechanisms of action. Such convergence provides a coherent explanation for the consistent cardiometabolic benefits observed across the GLP-1RA drug class and establishes a basis for evaluating differences in their clinical effects on oxidative biomarkers and organ protection [115,117].

### 7.3. Clinical Evidence for Improvements in Oxidative Biomarkers

An increasing amount of clinical evidence demonstrates that GLP-1RAs have beneficial metabolic effects and measurably improve systemic oxidative stress. Trials involving individuals with obesity and type 2 diabetes have consistently reported reductions in lipid peroxidation markers and enhanced antioxidant capacity following treatment [172,173]. The most reproducible findings include lower circulating concentrations of MDA, oxidised LDL and 8-isoprostaglandin F2α. These changes parallel weight reduction, improved insulin sensitivity and diminished inflammatory activity, indicating that redox improvements arise as part of broader metabolic recovery rather than as isolated pharmacological effects. Earlier clinical observations further support these metabolic benefits, with Du et al. (2014) [183] showing that GLP-1RAs improve glycaemic control, insulin sensitivity and cardiovascular risk profiles, providing a plausible biological basis for later reports of reduced oxidative injury. More recent data confirm sustained improvements in metabolic status and circulating oxidative biomarkers during prolonged GLP-1RA therapy, with additional studies demonstrating shifts in systemic redox balance towards a less oxidative state and enhanced endogenous antioxidant capacity [184]. Thus, the clinical literature indicates that the molecular actions of GLP-1 signalling are reflected in quantifiable reductions in oxidative stress and improvements in redox homeostasis.

### 7.4. Cardiovascular and Neuroprotective Actions of GLP-1RAs

The antioxidant properties of GLP-1RAs extend beyond classical metabolic tissues to the cardiovascular and nervous systems, where oxidative stress is widely recognised as a significant factor in disease progression. Both experimental and clinical evidence suggest that GLP-1RAs preserve mitochondrial function, reduce inflammatory signalling and enhance cellular resilience in tissues that are particularly susceptible to chronic oxidative damage [169,170,175]. Within the cardiovascular system, activation of the GLP-1 receptor improves endothelial integrity by reducing oxidative stress, limiting lipid peroxidation and preserving mitochondrial bioenergetics. Himmerich (2025) demonstrated that liraglutide, semaglutide and dulaglutide consistently reduce oxidative stress and apoptosis, whilst also improving endothelial function, even under conditions of chronic metabolic overload [175]. Similarly, studies [169] demonstrated that GLP-1RAs modulate oxidative stress, autophagy, and STAT3-related signalling pathways, thereby protecting vascular tissues against inflammation and oxidative injury. The vascular benefits are likely to reflect coordinated improvements in mitochondrial function, nitric oxide bioavailability, and intracellular antioxidant defences.

In addition to providing vascular protection, GLP-1RAs have robust neuroprotective effects that are closely linked to improvements in mitochondrial homeostasis and redox regulation. Activation of GLP-1Rs reduces oxidative stress, apoptosis and neuroinflammation while supporting neuronal survival, highlighting a direct link between incretin signalling and mitochondrial resilience [175]. Further evidence shows that GLP-1RA therapy disrupts the relationship between metabolic dysfunction and neuroinflammation by suppressing ROS-driven microglial activation and improving systemic inflammatory tone [170]. Therefore, these findings support the idea that oxidative stress plays a vital role in the connection between metabolic disease and neurodegeneration, and that GLP-1-based therapies can reduce this connection by regulating the balance between oxidation and inflammation.

There is also increasing evidence that improvements in redox balance contribute directly to the anti-inflammatory actions of GLP-1 receptor agonists. Comparative analyses suggest that semaglutide and tirzepatide have stronger anti-inflammatory properties than liraglutide and dulaglutide due to their greater metabolic efficacy and more significant impact on redox-sensitive signalling pathways [171,177]. Clinical studies have shown consistent reductions in inflammatory mediators, including C-reactive protein (CRP), tumour necrosis factor-α (TNF-α) and interleukin-6 (IL-6), alongside simultaneous improvements in oxidative biomarkers such as oxLDL and MDA [172,178]. The parallel changes in inflammatory and oxidative markers suggest that the two processes are closely interconnected during GLP-1RA therapy rather than being regulated independently. Of the incretin-based therapies currently available, tirzepatide represents a particularly advanced approach to dual-incretin modulation of metabolic and potentially redox-related pathways. By activating both GLP-1 and GIP receptors simultaneously, it produces greater improvements in body weight, insulin sensitivity, and inflammatory status than conventional GLP-1RAs [171,174]. As oxidative stress is closely linked to metabolic overload, the superior metabolic effects of tirzepatide may contribute to reductions in ROS generation and improved mitochondrial resilience. However, further research is required to establish whether dual incretin agonism provides direct antioxidant benefits beyond those achieved through improved metabolic control alone.

Thus, the available experimental and clinical evidence shows that GLP-1RAs offer integrated cardiovascular and neuroprotective benefits by improving mitochondrial function, reducing oxidative stress, and suppressing chronic inflammation simultaneously. Rather than acting through isolated molecular targets, GLP-1RAs appear to coordinate multiple interconnected signalling pathways that collectively enhance tissue resilience across diverse organ systems. Such pleiotropic actions provide a strong biological rationale for the expanding therapeutic applications of GLP-1RAs beyond glucose lowering and establish a basis for comparison with other cardiometabolic therapies that influence oxidative homeostasis [117].

An important emerging aspect of GLP-1RA therapy is its interaction with other cardiometabolic drug classes, particularly sodium–glucose cotransporter-2 (SGLT2) inhibitors. While both strategies reduce oxidative stress and improve metabolic outcomes, they do so through distinct biological mechanisms that are complementary rather than competitive [170,177,185]. GLP-1RAs primarily target mitochondrial function and redox-sensitive inflammatory pathways. They reduce ROS generation across multiple metabolically active tissues, including the liver, adipose tissue, skeletal muscle, vascular endothelium and the central nervous system, by improving mitochondrial efficiency, stabilising mitochondrial membrane potential and limiting electron leak from the respiratory chain [154,169,170,175]. These mitochondrial effects are accompanied by reduced lipid peroxidation, attenuation of inflammatory signalling and restoration of endothelial homeostasis.

In contrast, SGLT2 inhibitors mainly exert their antioxidant effects by correcting glucotoxicity and improving cellular energy metabolism. Reduced intracellular glucose overload decreases mitochondrial substrate pressure, lowers ROS generation and improves metabolic flexibility, particularly in renal and cardiac tissues [186,187]. Consequently, oxidative stress is primarily attenuated through improved substrate utilisation rather than direct modulation of mitochondrial stress-response pathways. The distinction between these mechanisms suggests that the two drug classes may provide complementary protection against oxidative injury. While GLP-1RAs regulate mitochondrial integrity, inflammatory signalling and antioxidant defences more broadly, SGLT2 inhibitors primarily reduce oxidative stress by improving glucose handling and cellular bioenergetics [188,189]. Such complementary mechanisms provide a strong biological rationale for combination therapy in patients with obesity and type 2 diabetes, as simultaneous correction of metabolic overload and mitochondrial dysfunction may produce additional reductions in oxidative stress and chronic inflammation [190].

Among the incretin-based therapies currently available, tirzepatide appears to produce particularly pronounced redox-related effects. Due to its dual GLP-1/GIP receptor agonism, it combines the mitochondrial and anti-inflammatory actions typical of GLP-1 receptor activation with improvements in insulin secretion, glucagon suppression, and systemic metabolic regulation [191]. Experimental studies indicate stronger reductions in inflammatory signalling than those observed with semaglutide, and large clinical trials consistently demonstrate substantial weight loss and marked improvements in glycaemic control—two major determinants of oxidative stress [171,174,192]. Taken together, the findings suggest that dual incretin therapy may represent an important further development in metabolically oriented strategies for targeting redox imbalance.

The available evidence suggests that GLP-1RAs act as systemic modulators of oxidative homeostasis, rather than merely being glucose-lowering agents. Their biological effects extend beyond glycaemic regulation, encompassing the preservation of mitochondrial integrity, the enhancement of endogenous antioxidant defences, the attenuation of inflammatory signalling, and the reduction in lipid peroxidation across multiple organs [154,165]. Clinical improvements in oxidative biomarkers, including decreased concentrations of malondialdehyde, oxidised LDL and 8-iso-PGF2α, are consistently accompanied by better insulin sensitivity, reduced systemic inflammation and improved endothelial function [172,173]. Thus, the evidence supports a model in which GLP-1RAs contribute to redox homeostasis by coordinating mitochondrial function, antioxidant signalling and inflammatory pathways. Rather than acting on a single molecular target, GLP-1RAs influence interconnected biological processes that collectively reduce ROS production, preserve mitochondrial quality, limit oxidative damage and improve tissue resilience. Such an integrated mode of action may help explain the broad cardiovascular, renal, hepatic and neuroprotective benefits reported in both experimental and clinical studies [193].

The accumulated evidence identifies redox regulation as an important mechanism contributing to the diverse therapeutic effects of GLP-1 receptor agonists. These agents interrupt the vicious cycle linking oxidative stress, metabolic dysfunction and progressive organ damage by simultaneously improving mitochondrial bioenergetics, strengthening antioxidant capacity and suppressing chronic inflammatory activation. This integrated, redox-centred framework provides a conceptual bridge to the final section of the review, which examines the clinical implications and future prospects of targeting GLP-1 signalling in metabolic diseases associated with oxidative stress [25,168].

## 8. Natural Bioactive Compounds Influencing GLP-1 and Oxidative Signalling

Pharmacological GLP-1RAs represent a significant development in the treatment of obesity, type 2 diabetes, and related metabolic complications. However, increasing attention is being given to nutritional strategies capable of modulating endogenous GLP-1 secretion and enhancing cellular resilience to oxidative stress. A growing body of evidence suggests that naturally occurring bioactive compounds, particularly polyphenols, terpenoids and alkaloids, can influence GLP-1 biology through several complementary mechanisms, including stimulation of intestinal enteroendocrine L-cells, preservation of incretin activity, and regulation of oxidative pathways involved in metabolic dysfunction [194,195]. Although these compounds do not reproduce the pharmacological potency of GLP-1 receptor agonists, they may converge on overlapping downstream pathways, such as mitochondrial protection, activation of endogenous antioxidant responses, and attenuation of chronic inflammatory signalling.

**Figure 3:**
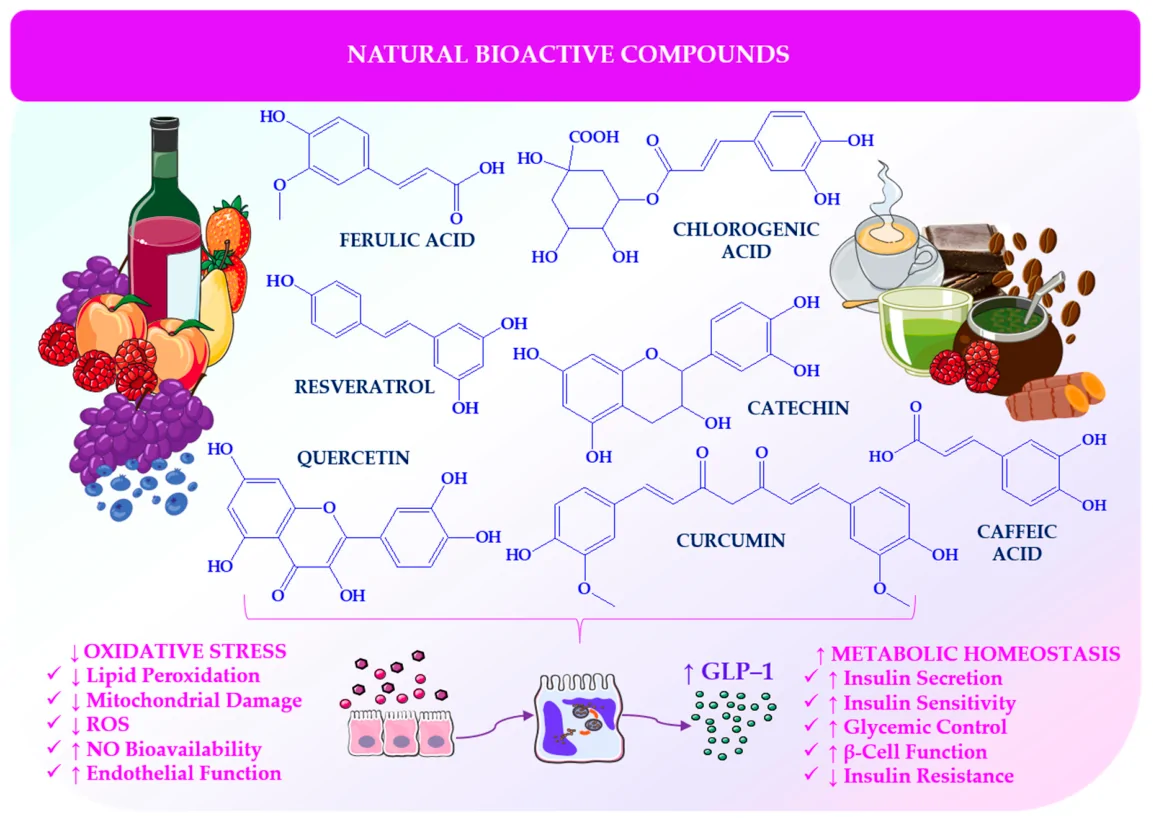
Natural bioactive compounds as modulators of GLP-1 signalling and metabolic immunity. Natural bioactive compounds increase GLP-1 secretion and activity and modulate redox pathways and mitochondrial function by activating antioxidant mechanisms and inhibiting pro-oxidant processes, thereby promoting improved metabolic homeostasis and cardiometabolic protection. Image provided by Servier Medical Art (https://smart.servier.com/), licenced under CC BY 4.0 (https://creativecommons.org/licences/by/4.0/, accessed on 31 July 2026). Abbreviations: GLP-1–glucagon-like peptide-1; NO–nitric oxide; ROS–reactive oxygen species.

Among naturally occurring compounds, polyphenols such as quercetin, resveratrol and epigallocatechin gallate (EGCG) have attracted considerable interest because of their ability to stimulate GLP-1 secretion and activate endogenous antioxidant pathways. Their effects appear to involve, at least in part, activation of Nrf2-dependent responses, enhancement of mitochondrial function, and limitation of ROS accumulation [196,197]. Terpenoids and alkaloids, including berberine and ginsenosides, similarly improve mitochondrial efficiency, reduce lipid peroxidation and attenuate inflammatory signalling, thereby influencing pathways that overlap with those activated by GLP-1 receptor stimulation [198,199,200,201]. In addition, suppression of pro-inflammatory mediators such as TNF-α and interleukin-6 may create a metabolic environment more favourable to incretin responsiveness and insulin sensitivity [202,203]. Natural bioactive compounds may therefore serve as complementary modulators of the GLP-1–mitochondrial–redox axis; however, their clinical effectiveness remains dependent on factors such as bioavailability, dosage, and the biological context in which they are administered [147]. Figure 3 illustrates how natural bioactive compounds modulate GLP-1 signalling and metabolic immunity. This is achieved by enhancing GLP-1 secretion and activity, as well as regulating redox pathways and mitochondrial function. The result is a reduction in oxidative and inflammatory processes, and an increase in metabolic homeostasis and cardiometabolic protection.

### 8.1. Resveratrol as a Modulator of GLP-1 Signalling and Mitochondrial Redox Balance

Resveratrol is among the most extensively studied polyphenols because of its ability to influence incretin biology and oxidative stress regulation. Previous studies have demonstrated that resveratrol may stimulate the secretion of the gut-derived incretin hormone GLP-1 [204], providing a potential link between dietary polyphenols and endogenous incretin regulation. Incretin-related activity is accompanied by activation of SIRT1/Sir2-dependent pathways, improved mitochondrial efficiency, and enhanced antioxidant capacity, all of which may contribute to the maintenance of metabolic homeostasis. In experimental models, resveratrol supplementation has been shown to increase portal GLP-1 and insulin concentrations, improve glucose tolerance, and reduce lipid peroxidation, indicating simultaneous effects on incretin signalling and oxidative balance [205]. Furthermore, studies in non-human primates have shown that resveratrol supplementation during a high-fat/high-sugar diet preserves pancreatic islet architecture and prevents β-cell dedifferentiation, suggesting a protective effect against metabolic stress-induced impairment of endocrine function [206].

The molecular basis of these responses involves the interplay between SIRT1 activation, mitochondrial regulation, and antioxidant defence pathways. By improving mitochondrial efficiency and reducing excessive ROS generation, resveratrol may limit oxidative damage affecting pancreatic β-cells, vascular tissues, and insulin-sensitive organs. Modulation of inflammatory pathways may provide an additional link between redox regulation and improved metabolic function [207,208]. Translation of these findings to human metabolic disease, however, requires careful consideration. Although preclinical studies consistently indicate antioxidant and metabolic benefits, clinical investigations have produced inconsistent results regarding the direct stimulation of GLP-1 secretion. In individuals with obesity or type 2 diabetes, resveratrol supplementation has not consistently altered postprandial GLP-1 responses, although improvements in selected metabolic parameters and inflammatory markers have been reported [209,210]. Accordingly, resveratrol is better regarded as a metabolic modulator that may support GLP-1-related pathways rather than as a substitute for pharmacological GLP-1 receptor agonists.

### 8.2. Polyphenols, Endothelial Protection and Glp-1-Related Vascular Regulation

Beyond their effects on incretin secretion, plant-derived polyphenols exert significant effects on vascular function through the regulation of NO bioavailability, endothelial function, and oxidative signalling. Their vascular actions are particularly relevant because endothelial dysfunction is a central feature of obesity, insulin resistance, and cardiovascular complications associated with type 2 diabetes [211,212]. Polyphenols enhance endothelial nitric oxide synthase (eNOS) activity, increasing NO production while reducing the oxidative degradation of endothelial signalling molecules, thereby contributing to greater vascular redox stability [211]. Limiting ROS-mediated impairment of endothelial function may consequently help preserve vascular responsiveness under metabolic stress. At the same time, flavonoids, anthocyanins and other phytochemicals can interact with enteroendocrine signalling pathways and promote GLP-1 release, linking intestinal nutrient sensing with vascular and metabolic regulation [33,213,214].

Black soybean seed coat polyphenols provide an illustrative example of such an interaction. In experimental models, these compounds have been shown to increase GLP-1 and cyclic adenosine monophosphate (cAMP) levels, promote eNOS phosphorylation, and enhance NO production, thereby linking incretin-related signalling with antioxidant vascular protection [215,216]. The mechanism reflects a broader capacity of dietary polyphenols to integrate intestinal hormone regulation with endothelial redox control. Human intervention studies provide further support for such a relationship. Consumption of polyphenol-rich foods, including jabuticaba juice, citrus flavonoids and anthocyanin-enriched products, has been associated with enhanced postprandial GLP-1 responses, reduced glucose excursions and increased total antioxidant capacity [217]. Improvements in these parameters have also been accompanied by reduced lipid peroxidation, greater glutathione availability, and enhanced endothelial signalling. Thus, the findings position polyphenols as potential nutritional regulators of the GLP-1–redox network, linking dietary bioactive compounds with metabolic and vascular resilience.

### 8.3. Curcumin, Catechins and Other Incretin-Active Antioxidants

In addition to resveratrol and flavonoid-rich preparations, several plant-derived bioactive compounds have been identified as modulators of both GLP-1 signalling and oxidative homeostasis. Curcumin and catechins are particularly relevant because they combine incretin-enhancing properties with direct antioxidant and anti-inflammatory activities [218,219]. Curcumin has been shown to influence intestinal L-cell function, enhance GLP-1 secretion, and improve glucose metabolism by reducing oxidative stress and inflammatory signalling [220,221]. At the mitochondrial level, curcumin may further improve cellular performance by limiting ROS generation, preserving mitochondrial membrane potential, and attenuating redox-sensitive inflammatory pathways [222]. The metabolic effects of curcumin therefore appear to reflect the convergence of incretin-dependent and redox-dependent mechanisms. By enhancing GLP-1 availability and reducing oxidative damage in insulin-sensitive tissues, curcumin improves glucose tolerance, reduces lipid accumulation, and protects pancreatic β-cells against oxidative injury [222,223]. Such effects are particularly relevant because persistent oxidative stress contributes substantially to β-cell dysfunction, impaired insulin secretion, and the progression of metabolic disease.

Catechins, particularly epigallocatechin gallate (EGCG), represent another group of polyphenols acting within the GLP-1–redox regulatory network. EGCG has been shown to stimulate GLP-1 release in enteroendocrine cell models while increasing antioxidant capacity and reducing lipid peroxidation [224,225]. Through activation of antioxidant defence mechanisms and modulation of mitochondrial function, catechins may reinforce endogenous incretin-mediated cytoprotection. They reduce ROS accumulation, improve cellular energy metabolism, and decrease inflammatory cytokine production, thereby supporting metabolic flexibility under conditions of nutrient excess [224,226]. Curcumin and catechins therefore illustrate the broader capacity of dietary bioactive compounds to influence metabolic homeostasis at several interconnected levels, including gut hormone secretion, mitochondrial function, and oxidative balance. Their ability to affect multiple components of the GLP-1 regulatory network suggests that they may complement pharmacological GLP-1RAs by providing additional antioxidant and cytoprotective activity.

### 8.4. Natural DPP-4 Inhibitors as Modulators of GLP-1 Stability and Oxidative Defence

An additional mechanism through which natural compounds influence GLP-1 activity involves inhibition of dipeptidyl peptidase-4 (DPP-4), the enzyme responsible for the rapid degradation of endogenous GLP-1. Reducing DPP-4 activity prolongs the half-life of active GLP-1, thereby enhancing incretin-mediated metabolic regulation and potentially improving oxidative defence mechanisms [227,228]. Several flavonoids, including myricetin and phloridzin, have demonstrated DPP-4 inhibitory activity in cellular and experimental models. Their activity has been associated with greater GLP-1 availability, improved glucose regulation, and attenuation of oxidative and inflammatory disturbances observed under diabetic conditions [229,230,231,232]. Importantly, such compounds may contribute to redox regulation in addition to preserving incretin signalling, through reductions in ROS generation and enhancement of endogenous antioxidant capacity. Mulberry-derived bioactive compounds provide another example of this dual activity. 1-Deoxynojirimycin (DNJ), cyanidin-3-glucoside (C3G) and cyanidin-3-rutinoside (C3R) have been shown to enhance glucokinase activity, increase intracellular ATP production and support insulin secretion under conditions of oxidative stress [233,234,235]. By protecting β-cells against ROS-mediated dysfunction, these compounds help maintain glucose-sensitive insulin secretion and establish a functional link between GLP-1-related signalling, cellular energy metabolism and antioxidant defence.

The convergence of DPP-4 inhibition and antioxidant activity is particularly relevant because it indicates that natural bioactives may preserve endogenous incretin activity while simultaneously reducing the oxidative burden associated with metabolic deterioration. Plant-derived DPP-4 inhibitors could therefore contribute to GLP-1-mediated metabolic regulation, particularly under conditions characterised by chronic inflammation, mitochondrial dysfunction and impaired redox balance [236,237]. Available evidence indicates that natural bioactive compounds can influence GLP-1 biology through several interconnected processes, including stimulation of GLP-1 secretion, inhibition of its degradation, enhancement of receptor-related signalling, and improvement in mitochondrial antioxidant capacity. Such activities are particularly evident in polyphenol-rich foods and plant extracts, which can regulate gut hormone secretion, endothelial function, and cellular redox status simultaneously [194].

Human intervention studies provide additional support for the potential physiological relevance of these interactions. Consumption of polyphenol-rich foods and beverages, including jabuticaba juice, citrus flavonoids, bilberry extracts and anthocyanin-rich products, has been associated with higher postprandial GLP-1 levels, improved glucose responses and increased total antioxidant capacity [217,238,239,240]. Dietary polyphenols may therefore influence physiological pathways also targeted by pharmacological incretin-based therapies, although their effects are generally less potent and more dependent on dietary context and bioavailability. Black soybean-derived polyphenols further illustrate how natural compounds can combine incretin-related and vascular protective effects. They increase GLP-1 and cAMP levels, promote endothelial eNOS phosphorylation, and enhance nitric oxide production, thereby linking antioxidant activity with GLP-1-dependent vascular regulation [241]. Thus, such observations support the view that the GLP-1–redox axis represents a coordinated physiological network connecting intestinal nutrient sensing, vascular homeostasis, and systemic metabolic regulation.

Natural bioactive compounds therefore appear capable of complementing pharmacological GLP-1RAs by targeting overlapping biological processes, including mitochondrial protection, antioxidant defence, suppression of inflammatory signalling, and preservation of β-cell function. Although their clinical efficacy requires further investigation, particularly with respect to bioavailability, dose optimisation and long-term effects, these compounds represent a biologically plausible nutritional strategy for supporting GLP-1-associated metabolic resilience [147,194].

### 8.5. Potential Synergy Between Natural Bioactive Compounds and Pharmacological GLP-1RAs

The considerable overlap between the actions of natural bioactive compounds and pharmacological GLP-1RAs raises the possibility of complementary interactions within the GLP-1–redox regulatory network. Although GLP-1RAs such as liraglutide, semaglutide, dulaglutide and tirzepatide exert potent incretin-mediated effects, natural compounds may influence oxidative and inflammatory pathways that contribute to metabolic dysfunction through complementary mechanisms [33,213,214]. Pharmacological GLP-1R activation improves mitochondrial efficiency, enhances antioxidant responses, reduces ROS formation and suppresses inflammatory signalling through pathways involving Nrf2, SIRT1, AMPK and other redox-sensitive regulators. Polyphenol-rich bioactives may reinforce several of these responses by increasing endogenous antioxidant capacity, improving mitochondrial function, and supporting intestinal GLP-1 secretion [195,196,197]. Combining pharmacological and nutritional strategies could therefore target distinct but interconnected components of metabolic dysfunction, integrating incretin signalling with redox regulation.

Experimental evidence provides preliminary support for such an approach. Co-administration of liraglutide and resveratrol has been shown to improve antioxidant status, reduce apoptosis, and moderate inflammatory responses in models of oxidative injury, suggesting potential complementarity between GLP-1R activation and polyphenol-mediated cytoprotection [242]. Natural compounds may consequently improve the cellular environment in which GLP-1 signalling operates, rather than simply reproducing the effects of pharmacological agonists. Similarly, compounds with DPP-4 inhibitory activity may complement GLP-1RAs by preserving endogenous incretin activity while reducing oxidative stress. Flavonoids such as myricetin and phloridzin, together with mulberry-derived compounds including DNJ, C3G and C3R, have been shown to improve glucose metabolism while strengthening antioxidant defence mechanisms [229,230,231,232]. Such dual activity may be particularly relevant to type 2 diabetes, in which impaired incretin signalling, mitochondrial dysfunction, and excessive ROS generation contribute simultaneously to disease progression.

Table 2 summarises the principal natural bioactive compounds that influence GLP-1 secretion, receptor-related signalling and redox regulation. By integrating findings from cellular, animal and human studies, the table illustrates the convergence of dietary bioactives on mitochondrial protection, antioxidant defence, endothelial function and metabolic homeostasis.

The interaction between dietary bioactives and GLP-1 signalling is particularly relevant in the context of chronic metabolic inflammation. Obesity and type 2 diabetes are characterised by persistent oxidative stress, mitochondrial dysfunction, and activation of inflammatory pathways such as NF-κB and the NLRP3 inflammasome. Natural compounds capable of enhancing GLP-1 activity may therefore contribute to reducing the inflammatory burden associated with metabolic disease. Polyphenols, anthocyanins and flavonoids can regulate inflammatory mediators such as TNF-α and IL-6, as well as pathways associated with oxidative stress, thereby supporting insulin sensitivity and vascular function [254,255]. By reducing inflammatory pressure and improving redox homeostasis, such compounds may enhance GLP-1 responsiveness indirectly and support more efficient metabolic adaptation. Several plant-derived compounds also influence mitochondrial quality-control mechanisms, including mitochondrial biogenesis, antioxidant enzyme activity, and cellular energy metabolism [256]. Examples that converge with mitochondrial effects observed following GLP-1 receptor activation include resveratrol-mediated SIRT1 activation, curcumin-induced mitochondrial protection, and catechin-associated antioxidant responses [204,205,220,221,222,223,224,225,226].

Thus, the available evidence indicates that the biological effects of natural compounds and GLP-1RAs are not independent phenomena but components of a broader metabolic regulatory network. Both pharmacological and nutritional interventions can influence interconnected cellular processes, including reduction in ROS accumulation, preservation of mitochondrial integrity, improvement in endothelial function, and protection of insulin-producing β-cells. The convergence of these pathways provides a mechanistic basis for considering nutritional bioactives as potential adjuncts to GLP-1-based pharmacotherapy, while recognising that their clinical relevance and possible synergistic effects require confirmation in well-designed human studies.

## 9. Short-Chain Fatty Acids and Microbiota-Derived Metabolites as Regulators of GLP-1 Signalling and Redox Homeostasis

There is accumulating evidence that microbiota-derived metabolites, particularly short-chain fatty acids (SCFAs), are key molecular links between intestinal microbial activity, incretin secretion, and systemic redox regulation. SCFAs, including acetate, propionate and butyrate, are produced when dietary fibres are fermented by intestinal microorganisms and function as signalling molecules involved in metabolic homeostasis [257,258]. SCFAs activate free fatty acid receptors (FFAR2 and FFAR3) on enteroendocrine L-cells, thereby directly stimulating GLP-1 secretion and connecting microbial metabolism with host endocrine regulation of glucose metabolism and energy balance [259]. Beyond their incretin-related effects, SCFAs contribute to cellular redox homeostasis by improving mitochondrial efficiency, limiting excessive ROS production, and strengthening intestinal barrier function. Through these combined actions, they may reduce inflammation-related oxidative injury and promote a metabolic environment that supports insulin sensitivity and mitochondrial resilience [260,261]. SCFAs should therefore be regarded not merely as products of microbial fermentation, but as bioactive mediators that integrate gut microbiota activity with endocrine, metabolic and oxidative signalling pathways.

The capacity of dietary components to increase populations of SCFA-producing microorganisms represents an important mechanism through which natural compounds may regulate GLP-1 secretion. Recent studies have demonstrated that plant-derived polysaccharides can remodel the gut microbiota towards beneficial, SCFA-producing bacteria, with subsequent enhancement of incretin activity and metabolic function [262,263]. For example, Yang et al. (2025) showed that a water-soluble polysaccharide derived from finger citron improved metabolic disturbances in mice fed a high-fat diet and treated with streptozotocin by modulating the gut microbiota–GLP-1 pathway [264]. The treatment increased the abundance of SCFA-producing bacteria, improved intestinal barrier integrity, and elevated circulating GLP-1 concentrations, supporting a role for microbial metabolites as upstream regulators of incretin signalling. Similarly, *Achyranthes bidentata* polysaccharide (ABP) has been shown to increase the abundance of SCFA-producing bacterial genera, restore intestinal barrier function, and enhance GLP-1-mediated metabolic regulation [265]. ABP activates the GLP-1/GLP-1R/cAMP/PKA/CREB/INS signalling cascade, leading to improved insulin secretion and glucose control. Taken together, the data point to a common biological mechanism in which natural polysaccharides promote microbial fermentation, increase SCFA availability, and activate GLP-1-dependent pathways that support metabolic flexibility and oxidative stability [265].

The molecular basis of SCFA-mediated incretin regulation involves direct activation of G-protein-coupled receptors on enteroendocrine cells. Following stimulation of FFAR2/FFAR3, intracellular signalling events promote GLP-1 secretion and subsequent activation of GLP-1 receptor-dependent pathways in peripheral tissues. Zhou et al. (2025) demonstrated that SCFA-induced GLP-1 release activates the GLP-1/GLP-1R/cAMP/PKA/CREB/INS pathway, thereby enhancing pancreatic insulin secretion and improving glycaemic regulation [266]. A functional connection between microbial metabolism and host endocrine control is thereby established, with microbiota-derived metabolites stimulating incretin signalling and GLP-1 activation subsequently supporting mitochondrial function and reducing oxidative stress. Further evidence supports the role of microbiota-regulated metabolites in maintaining the GLP-1–redox axis. Zhu et al. (2025) demonstrated that bile-processed Rhizoma Coptidis—a traditional preparation involving the treatment of the herb with bile to alter its alkaloid composition—improves glucose metabolism by modifying the composition of gut microbes and enhancing SCFA pathways [267]. This metabolic improvement was accompanied by increased GLP-1 secretion and improved metabolic parameters, which supports the role of microbial metabolites in incretin biology and redox regulation.

Although SCFAs are among the most extensively studied classes of microbiota-derived metabolites, increasing evidence indicates that bile acids also function as important microbiota-regulated signalling molecules linking intestinal microbial composition with GLP-1 secretion and oxidative homeostasis. Primary bile acids synthesised in the liver undergo extensive microbial transformation in the intestine, generating secondary bile acids that regulate host metabolism through receptors such as the Takeda G-protein-coupled receptor 5 (TGR5) and the farnesoid X receptor (FXR) [268,269]. Activation of these pathways influences enteroendocrine function, promotes GLP-1 secretion, and contributes to the regulation of mitochondrial metabolism in intestinal and hepatic tissues. The interplay among SCFAs, bile acids and incretin signalling therefore forms an integrated microbiota–endocrine–metabolic network. SCFAs produced by microbial fermentation activate FFAR2/FFAR3 receptors on intestinal L-cells, whereas microbiota-modified bile acids stimulate TGR5-dependent pathways that further enhance GLP-1 release [270,271]. Through coordinated effects on insulin secretion, energy metabolism and inflammatory responses, these microbial metabolites contribute to glucose homeostasis. Importantly, both SCFAs and bile acid-derived signals may support oxidative balance by improving mitochondrial efficiency, reducing excessive ROS generation, and maintaining intestinal epithelial integrity [272].

Recent experimental studies further support the view that modulation of the gut microbiota–bile acid axis can indirectly influence GLP-1-dependent metabolic regulation. For example, authors demonstrated that fibroblast growth factor 21 improves insulin sensitivity by regulating the bile acid–gut microbiota axis [273]. The findings highlight bile acid signalling as an important endocrine mediator of metabolic adaptation. Similarly, Zhu et al. (2025) showed that Rhizoma Coptidis processed with bile alleviates type 2 diabetes by reshaping the composition of the gut microbiome and enhancing SCFA metabolism, which is associated with improved incretin-related responses [267]. Authors further demonstrated the importance of beneficial microbial taxa in regulating incretin secretion, reporting that *Bifidobacterium animalis* MN-Gup improves glucose metabolism by increasing favourable microbial populations and their metabolites [274]. Changes in the microbial community were positively associated with circulating GLP-1 and insulin concentrations, suggesting that selected probiotic strains may influence endocrine metabolism through microbiota-derived signalling molecules. Thus, SCFAs, bile acids and other microbial metabolites appear to function as coordinated regulators of GLP-1 secretion and redox homeostasis rather than as isolated components of metabolic regulation.

The metabolic consequences of microbiota-derived metabolite signalling are mediated, in part, by GLP-1-dependent regulation of oxidative and inflammatory pathways. GLP-1 signalling exerts direct antioxidant and cytoprotective effects, improving mitochondrial function and stabilising the mitochondrial membrane potential while reducing excessive ROS accumulation [155,189]. GLP-1 also suppresses redox-sensitive inflammatory pathways, including NF-κB activation and NLRP3 inflammasome signalling, thereby limiting chronic inflammation associated with metabolic dysfunction [164]. SCFAs and GLP-1 can consequently be viewed as components of a reciprocal metabolic circuit: microbial metabolites stimulate intestinal GLP-1 secretion, while GLP-1 signalling reinforces mitochondrial stability, antioxidant defences, and inflammatory control. Such bidirectional communication provides a plausible mechanistic explanation for the influence of gut microbiota alterations on systemic metabolic health and oxidative balance [275].

SCFA-mediated enhancement of GLP-1 secretion has been associated with reduced oxidative damage through mechanisms involving improved mitochondrial efficiency, decreased lipid peroxidation, and suppression of pro-inflammatory mediators, including TNF-α and interleukin-6 [276]. Studies [273] demonstrated that modulation of the bile acid–microbiota axis improves insulin sensitivity through mechanisms associated with metabolic redox regulation. Meanwhile, authors [275] emphasised the bidirectional communication between GLP-1 signalling and gut microbial composition. The reciprocal nature of this interaction may create a self-reinforcing metabolic loop in which microbial metabolites enhance incretin activity, GLP-1 signalling improves oxidative resilience, and improved redox conditions help maintain a favourable intestinal microbial environment. Such a model provides a broader framework for understanding how dietary components, probiotics, and microbiota-targeted interventions may complement GLP-1-based pharmacological therapies for metabolic diseases [276].

**Figure 4:**
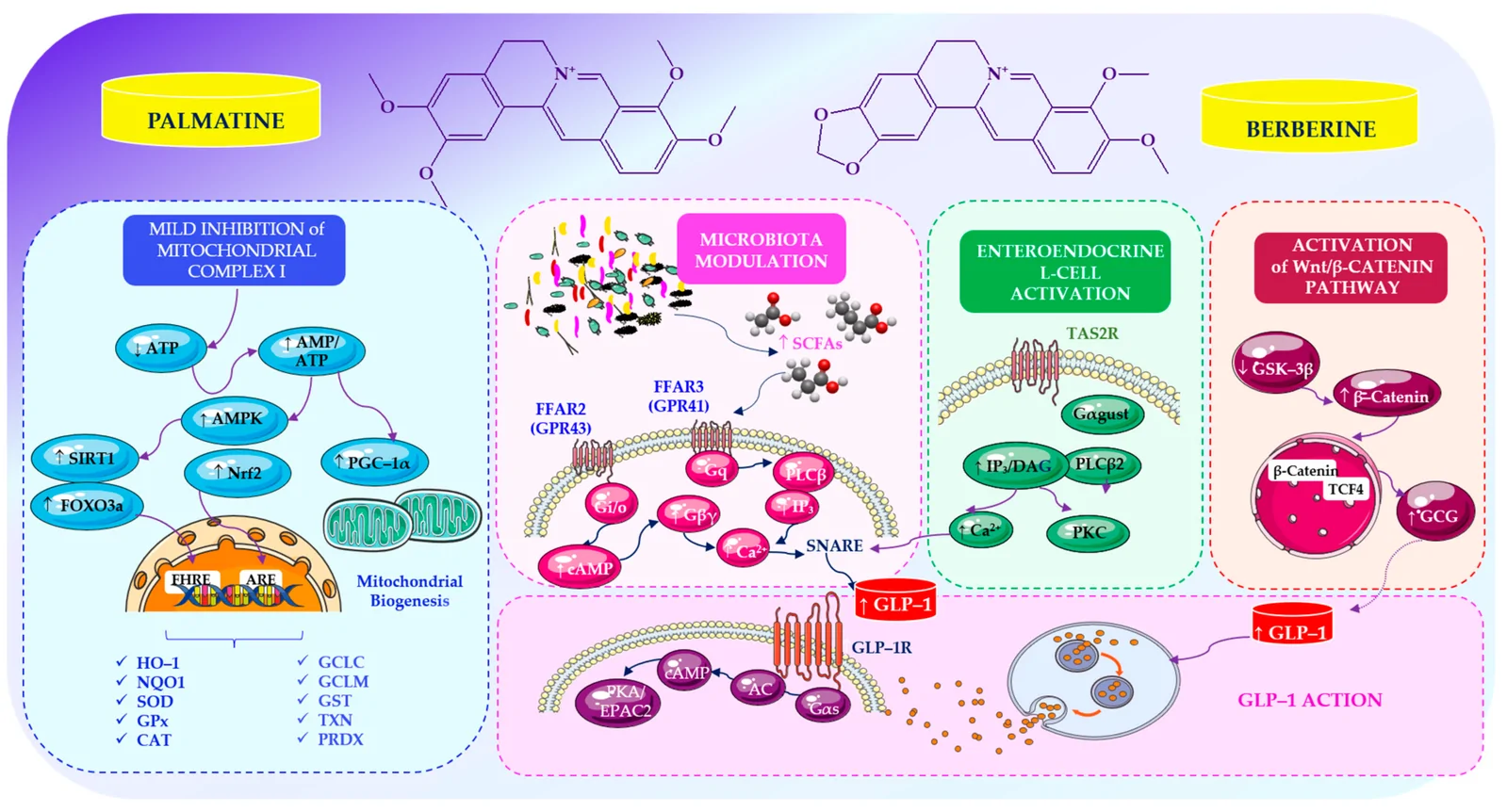
Mechanism of action of berberine leading to increased GLP-1 activity. Berberine increases GLP-1 secretion and synthesis by modulating the gut microbiota and SCFA production, activating FFAR2/FFAR3 and TAS2R receptors in enteroendocrine L cells, and activating the β-catenin/TCF4 and AMPK–SIRT1–Nrf2 pathways, thereby supporting proglucagon synthesis and mitochondrial protection. Released GLP-1 binds to the GLP-1R receptor on target cells, activating the Gs–adenylate cyclase–cAMP pathway, leading to PKA and EPAC2 activation, increased Ca^2+^ influx, enhanced insulin exocytosis, and improved glucose homeostasis. Image provided by Servier Medical Art (https://smart.servier.com/), licenced under CC BY 4.0 (https://creativecommons.org/licences/by/4.0/, accessed on 31 July 2026). Abbreviations: AC–adenylyl cyclase; AMP–adenosine monophosphate; AMPK–AMP-activated protein kinase; ARE–antioxidant response element; ATP–adenosine triphosphate; cAMP–cyclic adenosine monophosphate; CAT–catalase; DAG–diacylglycerol; EPAC2–exchange protein directly activated by cAMP 2; FFAR2 (GPR43)–free fatty acid receptor 2 (G protein-coupled receptor 43); FFAR3 (GPR41)–free fatty acid receptor 3 (G protein-coupled receptor 41); FHRE–forkhead response element; FOXO3a–forkhead box O3a; Gαgust–gustducin alpha subunit; Gαs–stimulatory G protein alpha subunit; Gβγ–G protein beta-gamma subunits; GCG–glucagon gene; GCLC–glutamate–cysteine ligase catalytic subunit; GCLM–glutamate–cysteine ligase modifier subunit; Gi/o–inhibitory G protein alpha subunit; GLP-1–glucagon-like peptide-1; GLP-1R–glucagon-like peptide-1 receptor; GPx–glutathione peroxidase; Gq–G protein alpha q subunit; GSK-3β–glycogen synthase kinase-3 beta; GST–glutathione S-transferase; HO-1–heme oxygenase-1; IP_3_–inositol 1,4,5-trisphosphate; NQO1–NAD(P)H:quinone oxidoreductase 1; Nrf2–nuclear factor erythroid 2-related factor 2; PGC-1α–peroxisome proliferator-activated receptor gamma coactivator-1 alpha; PKA–protein kinase A; PKC–protein kinase C; PLCβ–phospholipase C beta; PLCβ2–phospholipase C beta 2; PRDX–peroxiredoxin; SCFAs–short-chain fatty acids; SIRT1–sirtuin 1; SNARE–soluble N-ethylmaleimide-sensitive factor attachment protein receptor; SOD–superoxide dismutase; TCF4–transcription factor 4; TXN–thioredoxin; Wnt–wingless/integrated.

Among natural compounds capable of influencing the microbiota–GLP-1 axis, berberine is a well-characterised example of a bioactive molecule that combines microbial modulation, incretin regulation and redox control (Figure 4). It improves intestinal barrier function, modifies microbial composition, influences bile acid and SCFA profiles, and enhances GLP-1 secretion through multiple enteroendocrine mechanisms [277].

Recent studies suggest that berberine metabolites, such as berberrubine and palmatine, stimulate GLP-1 secretion by mitigating oxidative stress and enhancing mitochondrial function in intestinal L-cells [278]. Berberine also activates bitter taste receptor-dependent signalling and phospholipase C (PLC)-mediated pathways, thereby promoting GLP-1 release [279]. Other studies have shown that berberine increases GLP-1 production via β-catenin/TCF4 signalling [280] and restores impaired incretin secretion by protecting intestinal cells against mitochondrial dysfunction associated with diet-induced obesity [281]. Furthermore, berberine enhances GLP-1/GLP-1R/PKA signalling in intestinal L-cells and pancreatic α-cells, thereby contributing to improved β-cell function, insulin regulation, and metabolic stability [282]. Thus, the available evidence characterises berberine as a microbiota-responsive incretin modulator whose activity involves both microbial metabolic signals and intracellular redox regulation.

The metabolic activity of berberine extends beyond direct stimulation of incretin pathways and involves complex interactions among gut microbial composition, microbial metabolites, and host signalling pathways [277]. According to analysis [283], berberine enhances GLP-1 secretion through several complementary mechanisms, including increased SCFA production, improved intestinal barrier function, and the AMPK-dependent activation of metabolic pathways. By modifying microbial ecology and reducing intestinal inflammation, the compound may create conditions that favour enteroendocrine L-cell activity and sustained incretin responsiveness [283].

The bidirectional relationship between GLP-1 signalling and the gut microbiota further helps explain the broad metabolic effects of berberine. Authors [275] emphasised that microbial metabolites stimulate GLP-1 secretion, whereas GLP-1 signalling influences intestinal metabolic conditions, inflammatory status, and oxidative balance. Such reciprocal communication may contribute to berberine-mediated reductions in lipid peroxidation and inflammatory signalling, together with improved mitochondrial stability in metabolic disorders [275]. At the cellular level, enhancement of GLP-1-related pathways by berberine contributes to improved glucose handling and protection against oxidative stress. Berberine increases SCFA availability, activates AMPK signalling and improves mitochondrial efficiency, thereby supporting metabolic adaptation under conditions of insulin resistance and excessive ROS production. The combined effects indicate that berberine may function not only as a glucose-lowering compound but also as a regulator of the broader microbiota–incretin–redox network involved in metabolic homeostasis [278]. Importantly, the mechanisms described for berberine illustrate a broader principle observed with many natural bioactive compounds: their metabolic activity is often mediated through interactions among the intestinal microbiota, incretin hormones, and oxidative signalling pathways. As with the previously discussed polyphenols and plant-derived polysaccharides, berberine enhances endogenous GLP-1 activity while simultaneously improving antioxidant defence and reducing inflammatory stress [277,278].

Current evidence supports the existence of a complex SCFA–microbiota–GLP-1–redox axis integrating dietary components, microbial metabolism, and host endocrine regulation. Microbiota-derived metabolites, including SCFAs and bile acid derivatives, stimulate GLP-1 secretion through FFAR2/FFAR3-, TGR5- and related signalling pathways. GLP-1 activation subsequently enhances mitochondrial function, limits ROS accumulation, and suppresses chronic inflammatory responses [58,276]. Natural polysaccharides, polyphenols, berberine and microbiota-targeted interventions such as probiotics therefore have the potential to modulate multiple components of this regulatory network. By increasing populations of SCFA-producing microorganisms, improving intestinal barrier integrity and enhancing incretin responsiveness, such interventions may contribute to better glucose regulation and metabolic flexibility [147,212,262,263,277].

The convergence between microbiota-derived signalling and GLP-1-mediated antioxidant pathways also helps explain the complementary effects observed between dietary bioactive compounds and pharmacological GLP-1RAs. Although natural compounds generally lack the potency and pharmacological specificity of GLP-1RAs, they can influence overlapping biological processes, including mitochondrial protection, reduction in oxidative stress and attenuation of inflammatory signalling [275]. Accordingly, modulation of the gut microbiota–GLP-1–redox axis represents an emerging strategy for supporting metabolic resilience in type 2 diabetes and associated cardiometabolic disorders. Combining regulation of the microbial ecosystem with incretin enhancement and antioxidant protection may provide an additional therapeutic avenue for restoring metabolic homeostasis.

## 10. Plant Extracts and Bioactive Peptides Affect GLP-1 Pathways

Natural, plant-derived compounds and bioactive peptides can regulate endogenous GLP-1 activity via multiple, complementary mechanisms. These mechanisms include direct receptor-mediated signalling, modulation of the gut microbiota, and improvement in the intestinal redox environment [194,195]. Rather than acting through a single pathway, plant-derived bioactives may stimulate GLP-1 secretion directly through intestinal receptors, including bitter taste receptors, the Takeda G-protein-coupled receptor 5 (TGR5), and free fatty acid receptors (FFARs), or indirectly by reshaping the gut microbiota to promote SCFA production and bile acid signalling [284,285]. The convergence of endocrine, microbial, and oxidative pathways provides a plausible basis for the metabolic effects attributed to these compounds.

Several botanical compounds directly activate receptor systems involved in incretin secretion. For example, naturally occurring TGR5 agonists identified in *Fagonia cretica* activate bile acid-sensitive G-protein-coupled receptors on enteroendocrine L-cells, thereby enhancing GLP-1 biosynthesis and secretion [286]. Similarly, bitter taste receptor agonists present in *Momordica charantia* and the carotenoid-rich fruit *Momordica cochinchinensis* stimulate GLP-1 release via taste receptor-mediated chemosensory pathways [287,288]. At the same time, polysaccharides isolated from finger citron and *Achyranthes bidentata* can reshape the intestinal microbiota by increasing the abundance of SCFA-producing bacteria. The resulting microbial metabolites—primarily acetate, propionate, and butyrate—activate GPR41/GPR43 (FFAR3/FFAR2) receptors on enteroendocrine cells, further enhancing GLP-1 secretion [264,265]. Thus, the evidence indicates that natural compounds can amplify endogenous incretin signalling through complementary receptor-dependent and microbiota-mediated mechanisms.

In addition to stimulating GLP-1 secretion, many natural compounds enhance the efficiency and duration of GLP-1R signalling. Potential mechanisms include improved receptor responsiveness, enhancement of downstream cAMP–PKA signalling, and inhibition of DPP-4-mediated degradation of active incretins. Consequently, endogenous GLP-1 activity may be prolonged under conditions of metabolic and oxidative stress, thereby supporting glucose homeostasis and endocrine stability [105,289]. Fenugreek-derived peptides act as positive allosteric modulators of the GLP-1R, thereby enhancing receptor activation and downstream cAMP–PKA–CREB signalling [290]. Polyphenolic preparations, including mangiferin-rich mango leaf extract, exhibit GLP-1-like activity by improving incretin signalling and potentiating insulinotropic responses [291]. Other phytochemicals may preserve endogenous incretin activity through DPP-4 inhibition, thereby limiting the enzymatic degradation of GLP-1 and GIP. Examples include cocoa flavanols, black carrot anthocyanins, and phytochemical mixtures derived from tulsi, banyan, and jamun [289,292,293,294]. By increasing incretin bioavailability and prolonging GLP-1-related signalling, such mechanisms provide a potential basis for complementary interactions with pharmacological GLP-1 receptor agonists.

Another important aspect of plant-derived bioactives is their ability to reinforce redox homeostasis, creating a metabolic environment that favours efficient GLP-1 signalling [194,195]. Many extracts have potent antioxidant and anti-inflammatory properties, reducing ROS formation, preserving mitochondrial function, and suppressing the activation of redox-sensitive inflammatory pathways [295,296]. Improved redox control may, in turn, increase cellular resilience and support endogenous incretin signalling during chronic metabolic stress. For example, curcumin attenuates oxidative stress, improves mitochondrial stability, and enhances GLP-1-associated insulin signalling [221]. Mushroom extracts derived from *Coprinus comatus* increase GLP-1 and reduced glutathione (GSH) concentrations, thereby decreasing ROS formation and improving glycaemic control [297]. Furthermore, combined administration of *Mucuna pruriens* and *Lactobacillus rhamnosus* extracts modulates the GLP-1/Nrf2/NF-κB signalling axis, increasing SCFA production and suppressing pro-inflammatory cytokine expression [298]. Thus, the available evidence indicates that antioxidant phytochemicals may influence GLP-1 biology by stimulating incretin secretion, supporting mitochondrial function, preserving intestinal barrier integrity, and limiting chronic inflammation. Such redox-protective effects overlap with several mechanisms attributed to pharmacological GLP-1RAs and may therefore help create a cellular environment in which GLP-1RA therapy can exert its metabolic effects more effectively [25,65].

Table 3 summarises the main types of plant-derived bioactive compounds and peptides that influence GLP-1 biology via distinct but interconnected molecular mechanisms. Despite their structural diversity, these natural products act on several key targets, including the stimulation of endogenous GLP-1 secretion, activation of GLP-1 receptor signalling, inhibition of DPP-4 activity, modulation of gut microbiota-derived metabolites, and attenuation of oxidative stress. Thus, the available evidence indicates that phytochemicals regulate the incretin system through interconnected endocrine, metabolic, microbial, and redox pathways rather than through a single molecular target.

Thus, plant extracts and bioactive peptides appear to influence GLP-1 biology through a combination of receptor activation, gut microbiota modulation, preservation of endogenous incretin activity, and reinforcement of antioxidant defences. Rather than functioning as isolated metabolic regulators, these compounds may integrate endocrine, microbial, mitochondrial, and redox signalling into a coordinated response that supports glucose homeostasis, preserves mitochondrial function, and limits chronic inflammation [194,195,295,296]. The convergence of these mechanisms provides a biological rationale for considering selected plant-derived bioactives as complementary components of GLP-1 receptor agonist-based strategies for the prevention and management of metabolic disorders.

## 11. Tissue-Specific Effects of GLP-1 on Oxidative Signalling

GLP-1 integrates metabolic, inflammatory and redox cues in a highly tissue-dependent manner. Consequently, its downstream effects vary across adipose tissue, skeletal muscle, the liver, immune cells and the brain. Such differences reflect variation in receptor density, mitochondrial architecture, adipokine and cytokine environments, as well as local oxidative demands. Tissue specificity may therefore explain why natural compounds that enhance endogenous GLP-1 secretion, potentiate GLP-1R signalling and stabilise redox pathways can complement GLP-1RA therapy by providing organ-specific benefits [170,194,195].

### 11.1. Adipose Tissue and Effects of GLP-1 on ROS Formation, Adipokine Release and Inflammatory Tone

GLP-1 is increasingly recognised as a key regulator of oxidative signalling in adipose tissue. Within this metabolically active organ, it integrates metabolic, inflammatory, and endocrine pathways to restore redox homeostasis [65]. Adipose tissue is also an active endocrine and immunometabolic organ that contributes substantially to systemic oxidative stress in obesity and type 2 diabetes [52]. Excess adiposity promotes mitochondrial dysfunction, activation of NADPH oxidases, lipid peroxidation, and excessive ROS production, alongside chronic low-grade inflammation and dysregulation of adipokine secretion [307]. GLP-1RAs counteract many of these pathological processes by reducing oxidative injury, improving mitochondrial function and restoring metabolic homeostasis within adipose tissue [117].

**Figure 5:**
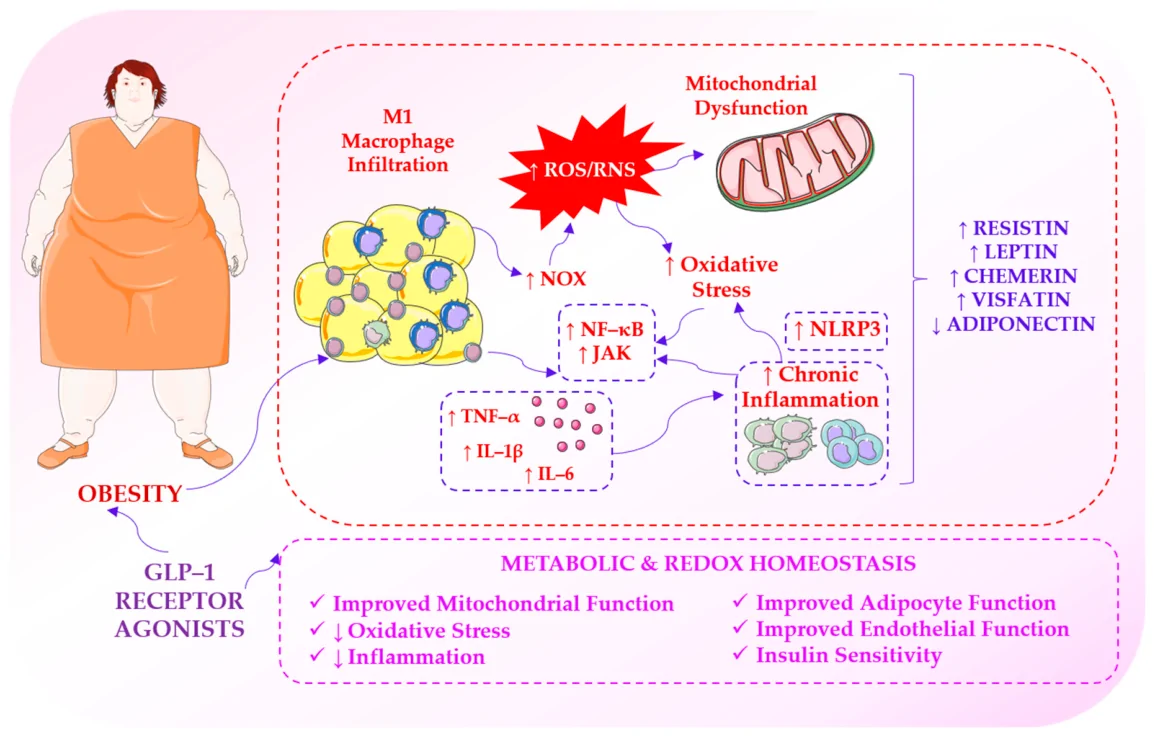
The role of GLP-1 receptor agonists in regulating oxidative stress and the inflammatory response in adipose tissue. Activation of the GLP-1 receptor reduces reactive oxygen species (ROS) production, improves mitochondrial function, inhibits inflammatory pathways (NF-κB, JNK, and NLRP3), modulates adipokine secretion, and promotes macrophage polarisation toward an anti-inflammatory phenotype. These effects lead to the restoration of redox homeostasis in adipose tissue, improved adipocyte function, increased insulin sensitivity, and improved metabolic homeostasis. Image provided by Servier Medical Art (https://smart.servier.com/), licenced under CC BY 4.0 (https://creativecommons.org/licences/by/4.0/, accessed on 31 July 2026). Abbreviations: GLP-1–glucagon-like peptide-1; IL-1β–interleukin-1 beta; IL-6–interleukin-6; JAK–Janus kinase; NF-κB–nuclear factor kappa B; NLRP3–NOD-, LRR- and pyrin domain-containing protein 3; NOX–NADPH oxidase; RNS–reactive nitrogen species; ROS–reactive oxygen species; TNF-α–tumour necrosis factor alpha.

Consistent findings from recent experimental and clinical studies demonstrate that GLP-1 signalling attenuates oxidative stress by reducing ROS generation, limiting inflammatory activation, and improving adipocyte function [52]. Exendin-4 decreases adipose tissue inflammation and extracellular matrix remodelling, thereby preserving adipose tissue architecture and metabolic function [308]. Similarly, semaglutide remodels both visceral and subcutaneous adipose tissue by reducing oxidative stress markers and restoring the expression of genes involved in mitochondrial metabolism and lipid handling [309]. Long-acting GLP-1 analogues, including E(X)-4(2)-Fc, further suppress leptin expression while improving metabolic signalling. Together, the findings provide additional evidence that GLP-1 directly modulates adipose endocrine activity [310]. Thus, GLP-1 signalling appears to restore adipocyte redox balance through coordinated improvements in mitochondrial function and metabolic signalling (Figure 5).

The beneficial effects of GLP-1 are closely linked to its ability to normalise adipokine secretion. Obesity disrupts adipokine balance by increasing pro-inflammatory adipokines such as leptin, resistin and chemerin, while reducing anti-inflammatory adipokines such as adiponectin. The resulting imbalance amplifies oxidative stress, insulin resistance and chronic inflammation [46,311]. Activation of the GLP-1R r partially reverses this profile by decreasing pro-inflammatory adipokines and restoring adiponectin production, thereby improving insulin sensitivity and reducing oxidative burden [141]. Regulation of adipokine secretion may therefore represent an important mechanism through which GLP-1 provides systemic metabolic protection beyond glucose control. Consistent associations have also been reported in obesity-related disorders such as psoriasis, heart failure with preserved ejection fraction (HFpEF) and endometriosis, where adipokine dysregulation contributes to oxidative tissue injury and inflammatory progression [312,313,314].

In addition to its role in endocrine regulation, GLP-1 also modulates immune signalling within adipose tissue [166]. Obesity promotes the infiltration of macrophages, as well as the activation of NF-κB, JNK and NLRP3 inflammasome pathways, thereby increasing ROS production and sustaining chronic inflammation [315]. GLP-1RAs suppress redox-sensitive inflammatory signalling, shift macrophage polarisation towards an anti-inflammatory phenotype, and reduce cytokine production, ultimately supporting the restoration of oxidative homeostasis [166]. Furthermore, emerging evidence indicates that immunometabolic interactions within adipose tissue contribute to the pathogenesis of numerous inflammatory and rheumatological disorders. Such observations reinforce the concept that GLP-1 regulates both oxidative stress and innate immune responses through interconnected mechanisms [316]. Importantly, the antioxidant actions of GLP-1 exhibit clear depot-specific characteristics. Visceral adipose tissue (VAT) displays greater mitochondrial dysfunction, oxidative stress, and inflammatory activity than subcutaneous adipose tissue (SAT) and appears to be particularly responsive to GLP-1-based interventions [317]. GLP-1RAs preferentially improve VAT by reducing ROS production, suppressing inflammatory adipokines and promoting mitochondrial biogenesis. In contrast, SAT predominantly demonstrates improvements in lipid metabolism and oxidative balance [189]. The distinct responses of VAT and SAT indicate that GLP-1 regulates adipose biology in a tissue-specific manner, contributing to the restoration of systemic redox homeostasis and metabolic resilience.

### 11.2. GLP-1 and Oxidative Signalling in Skeletal Muscle

Skeletal muscle is one of the main metabolic targets of GLP-1 signalling due to its key role in glucose disposal, lipid oxidation, and whole-body energy homeostasis [318]. Accumulating evidence indicates that GLP-1 has important regulatory effects on skeletal muscle oxidative metabolism, improving mitochondrial function, attenuating oxidative stress and enhancing insulin responsiveness [319,320]. In obesity and type 2 diabetes, skeletal muscle is particularly susceptible to chronic inflammation, mitochondrial dysfunction, and impaired substrate utilisation, all of which contribute to insulin resistance [321]. Under such conditions, GLP-1RAs can enhance mitochondrial biogenesis, improve oxidative phosphorylation, reduce excessive ROS generation, and restore metabolic flexibility. The resulting adaptations support improved insulin sensitivity and more efficient energy metabolism.

Beyond their direct effects on muscle cells, GLP-1RAs also improve the endocrine and inflammatory environment surrounding skeletal muscle [117]. Zhang et al. (2023) [322] demonstrated that GLP-1 normalises adiponectin signalling while reducing local inflammatory activity. The resulting changes indirectly enhance mitochondrial oxidative capacity, partly through the browning of neighbouring white adipose tissue. Further evidence from de Bem et al. (2018) [323] showed that combining physical exercise with plant extracts that modulate GLP-1, including *Euterpe oleracea* Mart. (açaí) extract, increases pAKT signalling and suppresses TNF-α expression in skeletal muscle. Thus, the findings indicate that lifestyle interventions and natural bioactive compounds may complement GLP-1 signalling by reinforcing mitochondrial function, improving redox balance, and enhancing metabolic resilience.

Table 4 summarises the main mitochondrial mechanisms that underlie oxidative metabolism and insulin sensitivity in skeletal muscle. Although these pathways have been described in different experimental settings, they converge on several biological processes that are also regulated by GLP-1 signalling. These processes include mitochondrial biogenesis, oxidative phosphorylation, ROS buffering, inflammatory cytokine signalling, and lipid handling. They illustrate how mitochondrial integrity contributes to metabolic flexibility and help explain why enhanced GLP-1 signalling can improve skeletal muscle function under conditions of metabolic stress.

Our understanding of how GLP-1 regulates skeletal muscle oxidative metabolism has evolved considerably over the past decade. Its biological actions clearly extend beyond glucose lowering, with increasing experimental and clinical evidence indicating that GLP-1 signalling enhances mitochondrial resilience, supports redox homeostasis, and suppresses inflammatory pathways that interfere with insulin action [324,325,326,327,328,329]. These observations are consistent with the findings of authors [322], who demonstrated that GLP-1-based interventions improve adiponectin signalling, reduce inflammatory tone, and enhance skeletal muscle oxidative capacity. Similarly, study [323] showed that plant extracts that modulate GLP-1 combined with exercise further enhanced pAKT activation while suppressing TNF-α expression. The convergence of pharmacological, nutritional and lifestyle interventions on mitochondrial and redox-sensitive pathways suggests that GLP-1 is an important regulator of skeletal muscle mitochondrial function. By promoting metabolic flexibility and insulin responsiveness, it may also protect muscle tissue against the oxidative damage associated with obesity and type 2 diabetes.

### 11.3. GLP-1 and Immune-Oxidative Crosstalk

There is growing evidence that GLP-1 is not only a metabolic hormone, but also an important regulator of immune function and oxidative homeostasis. Through its effects on nutrient sensing, it connects inflammatory and redox signalling pathways [44,53,105,189]. In addition to its well-established effects on glucose metabolism, activation of the GLP-1 receptor attenuates oxidative stress in both innate and adaptive immune cells. It also suppresses the production of pro-inflammatory cytokines and limits the activation of redox-sensitive signalling cascades, thereby contributing to systemic immunometabolic homeostasis [166]. Conversely, chronic low-grade inflammation associated with obesity and metabolic disease can profoundly alter immune cell function, promoting M1 macrophage polarisation, excessive ROS generation and activation of NF-κB, JNK and NLRP3 inflammasome pathways [330,331]. GLP-1RAs counteract the resulting inflammatory and oxidative imbalance by reducing oxidative stress, suppressing the release of inflammatory cytokines, and promoting a shift towards anti-inflammatory immune phenotypes [166]. GLP-1 signalling can therefore improve metabolic control while helping to restore immune homeostasis and interrupt the cycle linking oxidative stress with chronic inflammation.

Further experimental and clinical observations demonstrate that modulation of adipose tissue inflammation substantially contributes to the systemic antioxidant effects of GLP-1. For example, Rajamanickam et al. (2020) showed that helminth-induced immunomodulation reduces adipose-derived inflammatory mediators and improves systemic oxidative balance [332,333]. Although not directly focused on GLP-1, these findings provide an immunological framework that parallels several mechanisms attributed to GLP-1 signalling. Similarly, Ciancio et al. (2026) demonstrated that adipokine-driven inflammatory pathways contribute to oxidative stress in psoriasis [334], underscoring the significance of GLP-1-dependent regulation of immune–epidermal interactions. Thus, the evidence indicates that controlling inflammation in adipose tissue may favourably influence systemic redox status well beyond metabolic organs.

The immunomodulatory properties of GLP-1 appear to overlap with mechanisms observed in other inflammation-driven disorders. Authors reported that biological therapies used in the treatment of dermatological and rheumatological diseases modify adipokine secretion, mitochondrial function and oxidative stress [335]. Such findings highlight similarities with GLP-1-mediated immunometabolic regulation. Although these therapeutic approaches target distinct molecular pathways, they converge on shared biological processes, including suppression of inflammatory signalling, restoration of mitochondrial function, and reduction in ROS production.

Thus, the evidence identifies GLP-1 as an important mediator of immune–oxidative interactions. By regulating macrophage activity, inflammatory cytokine production, mitochondrial function and redox-sensitive signalling pathways simultaneously, GLP-1 integrates metabolic and immune responses into a coordinated protective network [166]. The multifaceted nature of this regulation broadens the therapeutic relevance of GLP-1RAs beyond glycaemic control, highlighting their potential to alleviate chronic inflammatory disorders characterised by persistent oxidative stress and immune dysregulation.

### 11.4. GLP-1 and Oxidative Signalling in the Liver

The liver is one of the main organs in which GLP-1 signalling has a significant impact, particularly in metabolic dysfunction-associated steatotic liver disease (MASLD/NAFLD), obesity, and type 2 diabetes, where it influences oxidative homeostasis, lipid metabolism, and inflammatory regulation [336]. Hepatic oxidative stress develops as a consequence of excessive lipid accumulation, mitochondrial dysfunction, chronic low-grade inflammation and increased ROS production, ultimately promoting insulin resistance and progressive liver injury [337]. There is increasing evidence that GLP-1RAs counteract these processes by improving mitochondrial function, reducing lipotoxicity, suppressing inflammatory signalling and restoring redox balance within hepatocytes [66].

Recent studies further demonstrate that the beneficial hepatic actions of GLP-1 extend beyond glucose regulation to involve multiple interconnected metabolic pathways. Visceral adiposity, intestinal dysbiosis, and adipokine imbalance substantially contribute to hepatic oxidative injury by activating inflammatory mediators, including BAFF/APRIL signalling pathways [336]. By improving lipid utilisation, reducing hepatic fat accumulation, and limiting ROS generation, GLP-1 signalling interrupts several of these pathogenic processes and promotes the restoration of hepatic metabolic homeostasis. The liver therefore represents an important site at which incretin signalling integrates endocrine, inflammatory, and oxidative responses [336].

Further evidence from pharmacological studies supports the protective role of incretin-based therapies against liver oxidative damage. For example, Ahn et al. (2017) demonstrated that the DPP-4 inhibitor gemigliptin reduced postprandial endotoxaemia and improved lipid oxidation following a high-fat meal [338]. It also attenuated metabolic disturbances associated with oxidative stress. Although DPP-4 inhibitors and GLP-1RAs act through distinct mechanisms, both therapeutic approaches enhance endogenous incretin activity and contribute to improved hepatic redox homeostasis. Such observations support the possibility that preserving GLP-1-related signalling may help limit oxidative liver injury in metabolic disorders. Additional clinical evidence comes from studies investigating metabolic surgery. Haluzíková et al. (2013) reported that sleeve gastrectomy significantly increased the secretion of endogenous GLP-1, together with the circulating fibroblast growth factors FGF-19 and FGF-21 [339]. The hormonal changes were accompanied by reduced systemic inflammation, improved lipid metabolism and enhanced hepatic metabolic adaptation. Thus, the findings provide further support for coordinated hepatoprotective signalling involving GLP-1, bile acid metabolism, mitochondrial function, and oxidative stress regulation.

The available evidence suggests that GLP-1 plays a key role in regulating hepatic oxidative signalling by integrating metabolic, inflammatory and mitochondrial pathways. By reducing ROS production, improving mitochondrial efficiency, lowering lipotoxicity and modulating inflammatory responses, GLP-1 helps to preserve liver function and may slow the progression of metabolic liver diseases. The organ-specific effects observed in the liver further support the therapeutic value of GLP-1-based interventions for modulating oxidative homeostasis in hepatometabolic disorders [336].

### 11.5. GLP-1 and Oxidative Stress in the Brain

The central nervous system is highly susceptible to oxidative damage due to its high oxygen consumption, high lipid content and relatively low antioxidant capacity. Disturbances in mitochondrial function, chronic neuroinflammation and excessive ROS production are recognised as key contributors to neurodegenerative and metabolic brain disorders [340]. There is increasing evidence that GLP-1 signalling protects the brain by reducing oxidative stress, preserving mitochondrial function and modulating inflammatory pathways [341]. One of the strongest lines of evidence for the neuroprotective role of GLP-1 comes from studies investigating Alzheimer’s disease. Zhang et al. (2024) demonstrated that semaglutide restores GLP-1 receptor expression, reduces amyloid burden and increases synaptophysin expression, a marker of synaptic integrity [342]. Concurrently, semaglutide decreases microglial activation, as evidenced by reduced expression of the ionised calcium-binding adaptor molecule 1 (Iba1) and the lysosomal macrophage marker CD68. The combined reduction in amyloid burden and microglial activation, together with preservation of synaptic markers, suggests that enhanced GLP-1 signalling may alleviate oxidative injury while suppressing neuroinflammatory processes involved in progressive neuronal degeneration [342].

GLP-1 plays an important role in metabolic brain dysfunction beyond neurodegenerative disorders through interactions with the gut–brain axis. Dysbiosis associated with obesity and metabolic syndrome reduces the production of SCFAs, impairs AMP-activated protein kinase (AMPK) signalling and promotes oxidative stress and neuroinflammation [326]. GLP-1 counteracts part of this pathological cascade by improving lipid homeostasis, restoring mitochondrial function and limiting ROS formation, thereby helping to preserve neuronal metabolism and cellular integrity. The close relationship between gut-derived metabolic signals and incretin activity further supports a role for GLP-1 in linking peripheral metabolic regulation with central nervous system homeostasis [170]. The interaction between GLP-1 and adipose tissue-derived endocrine signals provides an additional mechanism linking metabolic health to brain function. Adipokines such as leptin and adiponectin influence neuronal metabolism, mitochondrial activity, and oxidative signalling; their dysregulation contributes to cognitive impairment and chronic neuroinflammation. GLP-1-based therapies improve adipokine balance, thereby indirectly reducing oxidative stress and enhancing neuronal resilience [141]. Coordinated regulation of endocrine, inflammatory, and mitochondrial pathways therefore highlights the close relationship between peripheral metabolism and brain function.

Current evidence suggests that GLP-1 is an important regulator of oxidative signalling within the central nervous system. GLP-1 promotes neuronal survival and may slow the progression of both neurodegenerative and metabolic brain disorders by reducing ROS production, preserving mitochondrial integrity, limiting neuroinflammation, and improving metabolic communication along the gut–brain axis. Such tissue-specific effects reinforce the therapeutic potential of GLP-1RAs beyond glycaemic control, particularly with regard to neuroprotection and maintenance of cerebral redox homeostasis [341].

### 11.6. GLP-1 and Vascular Oxidative Signalling

The vascular endothelium is highly susceptible to oxidative stress, especially in the context of obesity, insulin resistance and chronic low-grade inflammation. Excessive production of ROS, endothelial mitochondrial dysfunction and reduced bioavailability of nitric oxide contribute to endothelial dysfunction, vascular remodelling and the progression of cardiometabolic disease [343]. There is increasing evidence that GLP-1 signalling protects the vasculature by restoring redox homeostasis, improving endothelial function and attenuating inflammatory activation [149]. One of the major mechanisms underlying these vascular benefits involves the regulation of adipose–vascular communication. Perivascular adipose tissue (PVAT) is now recognised as an active endocrine organ that releases adipokines and inflammatory mediators capable of promoting vascular ROS generation, endothelial dysfunction, and vascular remodelling [344]. GLP-1RAs counteract these processes by reducing leptin-driven oxidative stress, improving adipokine balance, and restoring NO/cGMP signalling, thereby preserving endothelial integrity and vascular function. Modulation of PVAT-derived signals therefore represents an important mechanism through which GLP-1 may limit oxidative vascular injury [150].

Further evidence suggests that GLP-1 has direct anti-inflammatory and antioxidant properties within the vascular wall. GLP-1 alleviates coronary microvascular inflammation and enhances vascular resilience by suppressing adipose-derived cytokine signalling, improving endothelial mitochondrial function, and reducing oxidative damage [189]. The same combination of metabolic, mitochondrial and anti-inflammatory effects has been observed across a broad spectrum of cardiometabolic disorders, supporting the view that GLP-1 integrates metabolic and vascular redox regulation [117]. Clinical studies further reinforce the vascular benefits of incretin-based therapies. For example, Shi et al. (2017) [345] demonstrated that GLP-1 analogues reduce intra-abdominal fat accumulation more effectively than acarbose, while also improving vascular oxidative markers. The findings indicate that vascular protection associated with GLP-1 extends beyond direct endothelial effects and is closely related to improvements in systemic metabolic status, adipose tissue function and inflammatory balance.

Thus, current evidence identifies GLP-1 as an important regulator of vascular oxidative signalling. By reducing ROS production, restoring endothelial nitric oxide signalling, improving mitochondrial function and modulating adipose–vascular crosstalk, GLP-1 enhances vascular resilience and helps preserve endothelial redox homeostasis. Such tissue-specific actions further broaden the therapeutic significance of GLP-1RAs beyond glycaemic control, highlighting their potential to prevent vascular complications associated with obesity, type 2 diabetes, and other cardiometabolic disorders [149].

## 12. Nutritional Modulation of GLP-1RA Efficacy in Anti-Inflammatory and Anabolic Mechanisms

The therapeutic effects of GLP-1RAs are increasingly recognised as being influenced by nutritional status, dietary composition and the metabolic environment. Beyond their well-established effects on appetite regulation and glycaemic control, GLP-1RAs modulate inflammatory pathways, mitochondrial function and tissue remodelling [117]. Nutritional strategies targeting the same biological processes may therefore enhance treatment efficacy, improve tolerability and minimise adverse metabolic consequences associated with prolonged weight loss, particularly the loss of lean body mass. One relevant nutritional approach is the use of anti-inflammatory and anabolic nutrients to support muscle preservation and metabolic flexibility. Varamini et al. (2026) [346] demonstrated that omega-3 polyunsaturated fatty acids have anti-inflammatory and anabolic properties in skeletal muscle. These properties enhance mTOR-p70S6K signalling and improve mitochondrial bioenergetics. This may help to reduce the decline in lean mass observed during GLP-1RA-associated weight loss. Similarly, flavonoids and anthocyanins may complement GLP-1-mediated improvements in insulin sensitivity by enhancing mitochondrial efficiency, reducing ROS formation, and attenuating inflammatory signalling [239]. Probiotics and prebiotics may provide an additional layer of support by modulating gut microbiota composition and increasing short-chain fatty acid production, which could also improve gastrointestinal tolerance during GLP-1RA therapy [142,347]. Taken together, targeted nutritional interventions may support treatment adherence while facilitating long-term metabolic adaptation.

Plant-derived bioactive compounds represent another important category of nutritional modulators of GLP-1-related pathways. While phytochemicals cannot substitute for pharmacological GLP-1 receptor agonists, growing evidence indicates that they can enhance endogenous incretin activity, improve insulin sensitivity and modulate downstream GLP-1 receptor signalling [194,195]. For example, Abiola et al. (2024) demonstrated that certain plant-derived compounds activate GLP-1R-related pathways, stimulate insulin secretion and enhance glucose tolerance [33]. Similarly, Akl and Ahmed (2025) showed that alkaloids derived from *Peganum harmala* enhance endogenous GLP-1 secretion and improve insulin responsiveness, suggesting potential interactions with incretin-mediated metabolic regulation [348]. In addition to their effects on incretin pathways, phytochemicals possess antioxidant properties that can reduce lipid peroxidation, support mitochondrial function and strengthen cellular defence mechanisms, thereby promoting metabolic resilience during GLP-1RA therapy [33].

The effectiveness and tolerability of GLP-1RA treatment are also strongly influenced by nutritional status and micronutrient availability. Appetite suppression induced by GLP-1RAs may reduce the intake of essential nutrients, such as protein, folate, and certain micronutrients. Inadequate intake may, in turn, contribute to fatigue, impaired muscle adaptation and reduced treatment tolerance [349]. Ruga et al. (2026) [350] emphasised that precision nutrition strategies, including adequate protein intake, omega-3 supplementation, and targeted micronutrient optimisation, may preserve lean mass, reduce gastrointestinal adverse effects, and improve adherence to long-term GLP-1RA therapy. Furthermore, Thomsen et al. (2025) [172] highlighted that treatment discontinuation is often linked to gastrointestinal intolerance and weight regain, underscoring the importance of nutritional support before, during and after pharmacological intervention to help maintain metabolic stability.

Nutritional support may be particularly relevant in light of the direct effects of GLP-1RAs on mitochondrial and cellular homeostasis. In addition to their effects on body composition and glucose metabolism, GLP-1RAs have important mitochondrial and cytoprotective properties mediated through the regulation of pathways related to oxidative stress [25,105]. For example, Tian et al. (2025) [148] demonstrated that GLP-1RAs can inhibit ferroptosis in diabetic kidney injury by activating β-Klotho and AMPK signalling. The resulting reduction in lipid peroxidation contributes to the restoration of iron homeostasis. Similarly, Cao et al. (2026) [351] showed that GLP-1RAs improve mitochondrial dynamics by regulating the balance between mitochondrial fission and fusion and reducing ROS accumulation in metabolic organs such as the kidney and liver. Thus, the evidence indicates that GLP-1-based therapies influence fundamental mechanisms of cellular adaptation, extending beyond glycaemic regulation to the preservation of mitochondrial integrity and redox equilibrium.

The systemic effects of GLP-1 signalling are also evident in reproductive, fibrotic and vascular tissues, where metabolic regulation intersects with inflammation and tissue remodelling. Voros et al. (2026) [352] reported that GLP-1RAs can improve ovarian function, reduce inflammation and modulate mitochondrial activity in women with polycystic ovary syndrome. Such effects suggest that the therapeutic actions of GLP-1RAs extend beyond weight reduction and glucose control. Yang et al. (2022) [353] demonstrated that activation of the GLP-1R attenuates fibrosis by reducing inflammatory cytokine production, improving mitochondrial function, and regulating extracellular matrix turnover. Furthermore, Wang et al. (2025) [354] showed that GLP-1-related improvements in autophagy and mitochondrial resilience may contribute to vascular protection by reducing oxidative injury. The broad tissue distribution of these effects supports the view that GLP-1-based therapies function as multi-organ regulators of inflammation, oxidative balance and tissue homeostasis.

Despite the promising results, careful consideration of individual variability is required when implementing nutritional adjuncts alongside GLP-1RA therapy. Differences in metabolic inflammation, gut microbiota composition, genetic background and incretin sensitivity may influence therapeutic responses [142]. Moreover, the bioavailability of phytochemicals and other natural compounds capable of modulating GLP-1 is strongly influenced by the food matrix, intestinal metabolism and microbiota-mediated transformation [33,142,147]. Long-term safety represents another important consideration, particularly for nutraceutical combinations that could affect nutrient intake, gastrointestinal function or the appetite-regulating effects of GLP-1RAs [355]. Well-designed clinical trials are therefore needed to determine optimal combinations and doses and to establish their long-term safety profiles. Against this background, GLP-1 has emerged as a central regulator of metabolic redox homeostasis, integrating mitochondrial function, inflammatory signalling and cellular antioxidant defence. Through activation of AMPK pathways, regulation of mitochondrial dynamics, improvement in nitric oxide–cGMP signalling and suppression of lipid peroxidation, GLP-1 contributes to the maintenance of oxidative balance in key metabolic tissues, including adipose tissue, the liver, the vasculature and the central nervous system [65,105].

The convergence of GLP-1RA pharmacology with nutritional modulation therefore represents an emerging therapeutic paradigm. GLP-1RAs reduce ROS formation, inhibit ferroptosis, regulate autophagy, and improve endothelial function, whereas dietary bioactive compounds such as polyphenols, anthocyanins, terpenoids, and omega-3 fatty acids can enhance antioxidant capacity, support mitochondrial biogenesis, and reduce inflammatory cytokine production. The complementary nature of these interventions may be reflected in changes in multiple metabolic health biomarkers, including MDA, 4-HNE, antioxidant enzymes such as SOD, GPx and catalase, adipokines, liver injury markers (ALT/AST), albuminuria and neuroinflammatory indicators [142,172,173,350]. Future therapeutic strategies combining GLP-1-based pharmacotherapy with precision nutrition, microbiome modulation and targeted antioxidant interventions may therefore provide a more comprehensive approach to obesity-, diabetes- and inflammation-related disorders. By coordinating endocrine, mitochondrial, immune and redox pathways across multiple organs, GLP-1 represents a unifying metabolic signal with considerable potential for the development of personalised therapeutic strategies.

## 13. Evidence Base and Weighting

**Figure 6:**
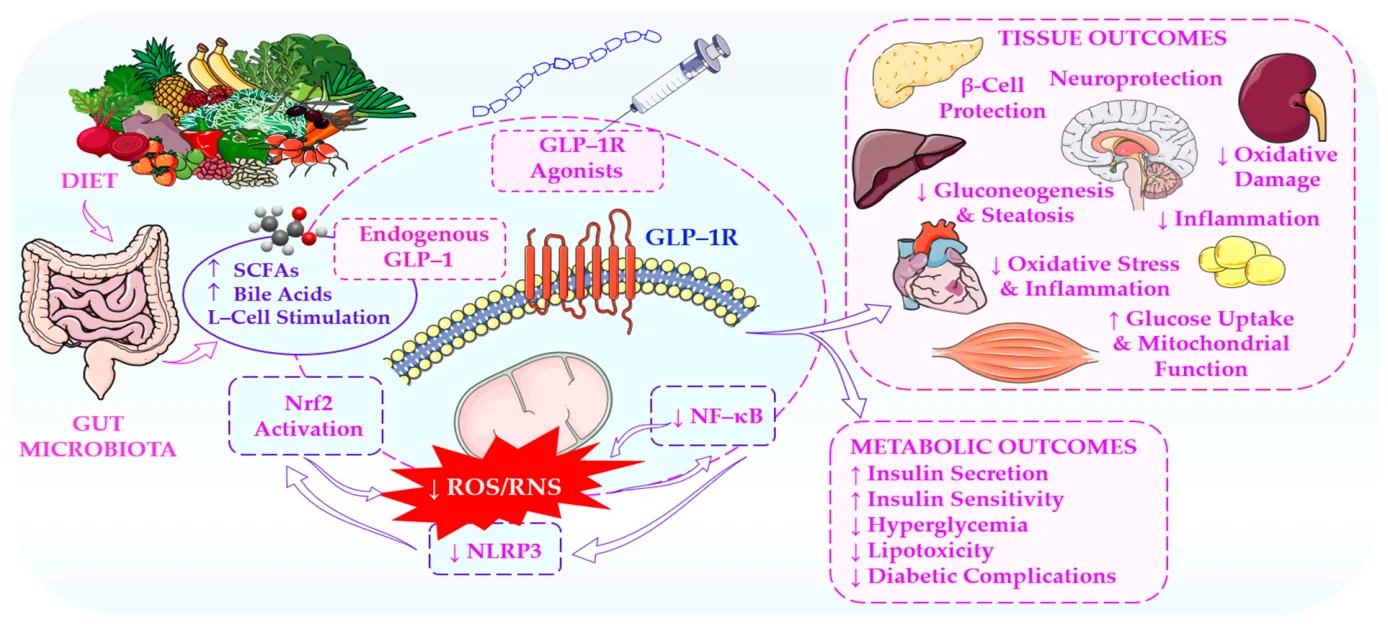
Integrated GLP-1/GLP-1R–mitochondrial–redox axis in metabolic homeostasis. An integrative framework illustrating the relationships between dietary and microbiota-derived signals, GLP-1 synthesis and secretion, mitochondrial function, redox regulation, metabolic signalling, and tissue-specific outcomes. The diagram shows how GLP-1 and GLP-1 receptor agonists interact with oxidative pathways to coordinate systemic metabolic responses in different organs. Image provided by Servier Medical Art (https://smart.servier.com/), licenced under CC BY 4.0 (https://creativecommons.org/licences/by/4.0/, accessed on 31 July 2026). Abbreviations: GLP-1—glucagon-like peptide-1; GLP-1R—glucagon-like peptide-1 receptor; NF-κB—nuclear factor kappa B; NLRP3—NOD-, LRR- and pyrin domain-containing protein 3; Nrf2—nuclear factor erythroid 2–related factor 2; SCFAs—short-chain fatty acids.

The evidence supporting the regulation of mitochondrial function and oxidative balance by GLP-1 is derived from cellular and ex vivo systems, animal models, and human clinical studies. Each level provides distinct information and is weighted according to its relevance for interpreting the axis: Diet/microbiota → GLP-1 → mitochondria → redox → metabolic signalling → tissue outcomes (Figure 6).

Studies performed in isolated cells and ex vivo preparations allow precise evaluation of the influence of GLP-1 on mitochondrial bioenergetics, ROS generation and antioxidant responses. These systems enable primary signalling pathways to be identified under controlled metabolic conditions. Key observations include suppression of mitochondrial ROS through LOX-1/GLP-1R signalling [107], interruption of hyperglycaemia-induced ROS persistence by GLP-1 cleavage products [110], preservation of astrocytic mitochondrial integrity via cAMP/PKA [111], restoration of mitochondrial dynamics through Epac/Akt [122], activation of SIRT1-linked antioxidant defence in renal tubular cells [127], regulation of steroidogenic mitochondrial function via FDX1 [131], modulation of mitochondrial fission–fusion balance through Drp1/Mfn2 [137], and autophagy-associated mitochondrial protection in retinal ganglion cells [139]. These findings define the cellular basis for GLP-1-mediated improvements in mitochondrial stability and oxidative balance.

Animal studies demonstrate that the cellular effects described above result in protection at the tissue and systemic levels. Evidence from cardiovascular, neurological and metabolic models indicates that GLP-1RAs enhance mitochondrial function, mitigate oxidative stress and minimise inflammatory damage in various organs. This includes enhanced cardiac mitochondrial bioenergetics in diabetic tissues, inhibition of ferroptosis in ischaemic brain injury, neuroprotection in Parkinson’s disease via Akt/JNK signalling, anti-apoptotic effects in cerebral ischaemia through PI3K/Akt/MAPK pathways, restoration of mitochondrial membrane potential in ageing cardiomyocytes, protection during hypoxia/reoxygenation injury, reduction in TNF-α-induced mitochondrial damage, endothelial protection in experimental hypertension via UCP2/GLP-1R, enhanced mitophagy in pulmonary arterial hypertension, AMPK/FoxO3a-linked improvements in myocardial I/R injury, SIRT3/FoxO3-dependent vascular regeneration, and SIRT1/SIRT3/AMPK.-associated antioxidant defence in myocardial injury [126]. These results confirm that GLP-1-related mitochondrial and redox effects operate at the level of whole tissues and organs.

Clinical studies confirm that GLP-1RAs have measurable systemic effects on oxidative stress. Studies investigating obesity and type 2 diabetes (T2D) have reported consistent reductions in lipid peroxidation markers, such as malondialdehyde (MDA), oxidised low-density lipoprotein (oxLDL) and 8-isoprostaglandin F2α, alongside improvements in antioxidant capacity [172,173]. Previous clinical studies have demonstrated the broad metabolic benefits of GLP-1RA therapy, including improved glycaemic control, insulin sensitivity, and cardiovascular risk profiles [183]. This provides a biological basis for subsequent observations of an improved redox status. Furthermore, data show sustained reductions in oxidative injury and enhanced endogenous antioxidant capacity during long-term GLP-1RA treatment [117,184], which is consistent with cellular and preclinical evidence of mitochondrial protection and reduced ROS generation [65,105]. These clinical findings confirm the translational relevance of GLP-1-mediated redox regulation.

Thus, cellular, preclinical, and clinical evidence provides a coherent foundation for the integrative framework developed in this review. Cellular studies identify primary pathways, animal models demonstrate tissue-level and systemic effects, and clinical data confirm relevance for human metabolic disease. This multi-layered evidence base supports the central concept that GLP-1 contributes to mitochondrial stability and oxidative balance across metabolically active tissues.

## 14. Future Directions

Future research should focus on refining our understanding of how nutritional signals, microbiota-derived metabolites and GLP-1 receptor activation converge to influence mitochondrial behaviour, cellular redox balance and downstream metabolic pathways. To clarify these links, a deeper analysis of mitochondrial dynamics, AMPK-linked metabolic regulation and redox-responsive transcription factors, such as Nrf2 and NF-κB, is required. This is especially important given that current evidence shows that GLP-1RAs affect ferroptosis, lipid peroxidation, mitochondrial stability and antioxidant enzyme activity. However, the intracellular sequence connecting these events remains insufficiently defined [117]. The gut microbiome is a second essential direction, as metabolites such as SCFAs, indoles and secondary bile acids may shape GLP-1 signalling by modifying mitochondrial efficiency, inflammatory tone and redox status; dysbiosis-related changes in AMPK activity, ROS formation and metabolite production may explain variability in responses to GLP-1RA therapy, highlighting the need for microbiome sequencing, metabolomics and detailed pharmacodynamic profiling to identify microbial signatures predictive of metabolic and oxidative outcomes [275]. Another priority is developing next-generation incretin-based therapies that can strengthen these interconnected processes by improving metabolic control and restoring redox balance through pathways involving AMPK activation, β-Klotho signalling, mitochondrial quality control, and suppression of lipid peroxidation [117,356]. Therefore, future therapeutic strategies should consider an individual’s metabolic phenotype, inflammatory status, microbiota composition, nutritional background and oxidative stress burden in order to achieve more effective long-term interventions for obesity, type 2 diabetes, metabolic dysfunction-associated steatotic liver disease and other disorders driven by oxidative imbalance [55,65,72,151]. This approach would reinforce the idea that GLP-1 acts at the intersection of endocrine regulation, mitochondrial function, immune responses and redox homeostasis.

## 15. Limitations

Despite growing evidence supporting the role of GLP-1 in regulating oxidative signalling, several important limitations currently restrict the translation of these findings into clinical practice. The available literature is characterised by substantial heterogeneity in study designs, experimental models, intervention strategies and patient phenotypes, making direct comparisons between studies difficult and limiting the identification of universal mechanisms. Furthermore, many studies investigating GLP-1–redox interactions concentrate on particular signalling pathways or specific tissues, whereas the systemic and multi-organ aspects of metabolic regulation remain insufficiently explored. Dias et al. (2025) [142] emphasised that nutritional interventions, including omega-3 fatty acids and bioactive compounds, can influence inflammation and cellular metabolism. However, most available studies involve short-term interventions only and provide limited information on long-term metabolic adaptation, sustained redox remodelling and the durability of clinical effects. Similarly, although microbiota-dependent regulation of GLP-1 secretion is increasingly recognised, the current evidence base largely comprises preclinical models, experiments and cross-sectional human studies, rather than long-term, controlled clinical trials [143,146,204,259,275]. Consequently, the causal relationships between gut dysbiosis, microbial metabolites, AMPK-dependent signalling, GLP-1 responsiveness and the regulation of oxidative stress remain incompletely defined.

Another important limitation concerns the tissue-specific effects of GLP-1 receptor agonists. While there is growing evidence that GLP-1RAs have protective effects against oxidative injury, such as inhibiting ferroptosis in diabetic kidney disease [148], improving vascular redox balance [150,165,189] and modulating hepatic inflammatory pathways associated with BAFF/APRIL signalling [356], much of this research is based on isolated cellular systems or animal models. The relevance of these observations to complex human metabolic disorders, in which the liver, adipose tissue, immune system, vasculature and central nervous system interact simultaneously, therefore requires further validation. The therapeutic potential of natural GLP-1-modulating compounds is also limited by several translational challenges. While phytochemicals, alkaloids, polyphenols and other plant-derived molecules can enhance GLP-1 secretion, activate GLP-1-related pathways or improve redox balance [195,196,211,212,216,236,237,241,243,294,295,296], their clinical application is limited by factors such as variable bioavailability, differences in absorption and metabolism, microbiota-dependent transformation, inconsistent dosing strategies and a lack of long-term safety data. Furthermore, the interaction between nutraceutical interventions and pharmacological GLP-1-based therapies requires systematic investigation to determine whether these approaches have additive, synergistic, or context-dependent effects.

Addressing these limitations will require an integrated approach combining multi-organ redox profiling, microbiome characterisation, metabolomics, pharmacodynamic analyses and long-term clinical monitoring. Such strategies are crucial for identifying which patients are most likely to benefit from GLP-1-based interventions, developing biomarkers capable of predicting therapeutic responses, and establishing evidence-based combinations of pharmacological and nutritional approaches. Thus, the current limitations highlight the need for studies that bridge molecular mechanisms, whole-body metabolic regulation and clinically relevant outcomes, thereby allowing the therapeutic potential of GLP-1-centred redox modulation to be defined more precisely.

## 16. Conclusions

This review compiles evidence from the fields of endocrinology, redox biology, microbiome science and nutritional biochemistry, demonstrating that GLP-1 acts as a regulator that connects nutrient-derived signals, microbiota activity, mitochondrial behaviour and cellular redox balance. GLP-1 signalling has been shown to influence pathways linked with metabolic adaptation in cellular, preclinical and clinical studies. These pathways include AMPK-related regulation, mitochondrial quality control, NLRP3 suppression, autophagy processes and protection from oxidative injury, such as ferroptosis. The reviewed data indicate that GLP-1 activity depends strongly on the surrounding metabolic environment, with microbiota-derived metabolites shaping incretin secretion, sensitivity and redox tone, while nutritional components such as omega-3 fatty acids, polyphenols, anthocyanins, probiotics, prebiotics and high-quality protein can reinforce GLP-1-related improvements in mitochondrial stability and antioxidant defence. Thus, these findings support the view of GLP-1 as a signalling node that links endocrine regulation with mitochondrial function, immune–metabolic communication and redox balance. This expands the therapeutic relevance of GLP-1 beyond glycaemic control, providing a foundation for future strategies combining pharmacological GLP-1 modulation with precision nutrition, microbiome-targeted approaches and personalised metabolic care.

## Figures and Tables

**Table 1:** Experimental and clinical evidence demonstrating GLP-1-mediated regulation of mitochondrial function, oxidative stress and inflammatory signalling.

Experimental Model/System	Principal Mechanism	Main Signalling Pathway(s)	Dominant Redox Mechanism	Key Finding	Reference
Cardiovascular tissues in type 2 diabetes	Improved mitochondrial bioenergetics	Mitochondrial oxidative metabolism	Reduced mitochondrial ROS production; improved mitochondrial bioenergetics	GLP-1RAs and SGLT2 inhibitors improve cardiac mitochondrial function and reduce oxidative stress	[132]
Human vascular smooth muscle cells	Regulation of mitochondrial ROS	LOX-1/GLP-1R	Reduced mitochondrial ROS generation	GLP-1 signalling suppresses mitochondrial ROS through LOX-1	[107]
Hyperglycaemia-exposed cells	Disruption of ROS feedback	GLP-1 cleavage products	Disruption of persistent ROS-generating feedback (“metabolic memory”)	GLP-1 metabolites interrupt hyperglycaemia-induced ROS persistence	[110]
Astrocytes (Alzheimer’s disease)	Mitochondrial preservation	cAMP/PKA	Reduced oxidative stress; preservation of mitochondrial integrity	Improved astrocytic mitochondrial function	[111]
Cardiomyocytes (IL-1β)	Protection from oxidative injury	GLP-1R/NF-κB	Reduced ROS production; suppression of inflammatory signalling	Liraglutide preserves mitochondrial membrane potential	[112]
Ischaemic brain injury	Ferroptosis inhibition	FoxO1/DRP1	Inhibition of ferroptosis; reduced lipid peroxidation	Semaglutide protects mitochondrial integrity	[121]
SH-SY5Y neuroblastoma cells	Restoration of mitochondrial dynamics	Epac/Akt	Reduced mitochondrial ROS; restoration of mitochondrial dynamics	Exendin-4 restores mitochondrial function	[122]
Diabetic vascular smooth muscle cells	Improved mitochondrial homeostasis	GLP-1/CREB/Adiponectin	Improved mitochondrial homeostasis and respiratory function	Improved vascular bioenergetics	[133]
H9c2 cardiomyoblasts (cisplatin)	Anti-apoptotic protection	p53/NF-κB	Reduced oxidative stress and mitochondrial apoptosis	Exendin-4 preserves mitochondria	[120]
Diabetic wound healing (EPCs)	Improved endothelial repair	NO signalling	Increased nitric oxide bioavailability; reduced mitochondrial ROS	Improved EPC function	[128]
Parkinson’s disease	Neuroprotection	Akt/JNK	Attenuation of mitochondrial stress and oxidative injury	Dual GLP-1/GIP agonism reduces oxidative injury	[134]
Renal tubular cells	Anti-lipotoxic protection	SIRT1	Activation of SIRT1-dependent antioxidant defence; reduced oxidative stress	Exenatide restores mitochondrial function	[127]
Cerebral ischaemia	Anti-apoptotic signalling	PI3K/Akt/MAPK	Activation of pro-survival signalling; reduced oxidative injury	Liraglutide protects neurons	[135]
α-PVP cognitive deficit	Brain mitochondrial protection	GLP-1R signalling	Reduced brain oxidative stress; improved mitochondrial function	Liraglutide improves cognition	[136]
PCOS	Regulation of steroidogenesis	FDX1	Improved mitochondrial bioenergetics and steroidogenic function	Improved ovarian mitochondrial function	[131]
Type 2 diabetes metabolic phenotypes	Regulation of mitochondrial dynamics	Drp1/Mfn2	Restoration of mitochondrial fission–fusion balance; reduced mitochondrial ROS	GLP-1 linked with mitochondrial dynamics	[137]
Ageing cardiomyocytes	Recovery of mitochondrial function	Cardiac GLP-1R	Reduced oxidative stress; recovery of mitochondrial membrane potential	Liraglutide restores mitochondrial membrane potential	[115]
H9c2 hypoxia/reoxygenation	Cardioprotection	Mitochondrial survival pathways	Reduced ROS generation; improved ATP production	Exendin-4 limits reperfusion injury	[124]
TNF-α cardiomyocytes	Anti-inflammatory protection	GLP-1R/ROS	Reduced mitochondrial oxidative injury and apoptosis	Reduced mitochondrial injury	[125]
Experimental hypertension	Endothelial protection	UCP2/GLP-1R	Improved endothelial redox balance; increased nitric oxide availability	Improved endothelial function	[138]
Retinal ganglion cells	Regulation of autophagy	Mitochondrial–autophagy pathway	Enhanced autophagic protection; reduced oxidative injury	Liraglutide protects retinal neurons	[139]
Pulmonary arterial hypertension	Regulation of mitochondrial turnover	DRP1/NOX/Beclin-1/LC3β	Enhanced mitophagy; reduced mitochondrial oxidative stress	Reduced mitochondrial stress	[114]
Myocardial I/R injury	Improved cardiac metabolism	AMPK/FoxO3a	Activation of AMPK signalling; improved mitochondrial bioenergetics	Enhanced post-ischaemic recovery	[140]
Endothelial progenitor cells	Vascular regeneration	SIRT3/FoxO3	Activation of SIRT3-dependent antioxidant pathways; reduced ROS	Improved vascular repair	[129]
Myocardial I/R injury	Enhanced antioxidant defence	SIRT1/SIRT3/AMPK	Enhanced endogenous antioxidant defence; preservation of mitochondrial function	Reduced mitochondrial injury	[126]
Leydig cells	Improved mitochondrial bioenergetics	GLP-1R signalling	Improved mitochondrial bioenergetics; reduced oxidative stress	Improved mitochondrial function	[130]
db/db mice	Ferroptosis inhibition	ROS/SOD2	Reduced ferroptosis; enhanced antioxidant enzyme activity (SOD2)	Improved cognitive performance	[123]

Abbreviations: Akt, protein kinase B; AMPK, AMP-activated protein kinase; ATP, adenosine triphosphate; Atg, autophagy-related protein; cAMP, cyclic adenosine monophosphate; CREB, cAMP response element-binding protein; DPP-4, dipeptidyl peptidase-4; DRP1, dynamin-related protein 1; EPCs, endothelial progenitor cells; Epac, exchange protein directly activated by cAMP; FDX1, ferredoxin 1; FoxO, Forkhead box O; GIP, glucose-dependent insulinotropic polypeptide; GLP-1, glucagon-like peptide-1; GLP-1R, glucagon-like peptide-1 receptor; GLP-1RA, glucagon-like peptide-1 receptor agonist; HMGB1, high-mobility group box 1; IL, interleukin; JNK, c-Jun N-terminal kinase; Keap1, Kelch-like ECH-associated protein 1; LC3β, microtubule-associated protein 1 light chain 3β; LOX-1, lectin-like oxidised LDL receptor-1; MAPK, mitogen-activated protein kinase; mtROS, mitochondrial reactive oxygen species; NF-κB, nuclear factor kappa B; NO, nitric oxide; NOX, NADPH oxidase; NRF1, nuclear respiratory factor 1; Nrf2, nuclear factor erythroid 2-related factor 2; PGC-1α, peroxisome proliferator-activated receptor gamma coactivator 1-alpha; PI3K, phosphoinositide 3-kinase; PKA, protein kinase A; ROS, reactive oxygen species; SGLT2i, sodium–glucose cotransporter-2 inhibitors; SIRT, sirtuin; TFAM, mitochondrial transcription factor A; TNF-α, tumour necrosis factor alpha; UCP2, uncoupling protein 2.

**Table 2:** Natural bioactive compounds regulating GLP-1 signalling and redox homeostasis: mechanisms, experimental evidence and metabolic outcomes.

Bioactive Compound/ Intervention	Experimental Model	Primary GLP-1-Related Mechanism	Redox and Mitochondrial Mechanisms	Main Metabolic Outcomes	Reference
Resveratrol	Rodent models of diabetes and metabolic dysfunction	Enhances intestinal GLP-1 secretion; improves incretin-mediated glucose regulation; influences GLP-1/GLP-2-related intestinal pathways	Activates SIRT1/Sir2 signalling; improves mitochondrial efficiency; reduces ROS formation and lipid peroxidation; enhances antioxidant defence	Improved glucose tolerance, increased portal GLP-1 and insulin concentrations, improved mitochondrial function	[205]
Resveratrol	Non-human primates fed high-fat/high-sugar diet	Modulates islet hormone regulation and GLP-1-related metabolic responses	Preserves mitochondrial function, enhances antioxidant capacity and protects pancreatic tissue from metabolic stress	Prevents β-cell dedifferentiation, improves islet morphology and limits metabolic deterioration	[206]
Resveratrol supplementation	Humans with obesity and type 2 diabetes	Effects on GLP-1 secretion remain inconsistent; may influence incretin responses indirectly	Reduces oxidative stress and inflammatory signalling; improves selected metabolic markers	Modest improvements in metabolic parameters, although GLP-1 effects are variable	[209,210]
Polyphenols (flavonoids, anthocyanins, resveratrol-rich compounds)	Endothelial dysfunction models and vascular studies	Indirect enhancement of incretin signalling; modulation of gut hormone secretion	Activation of eNOS/NO pathway; reduction in ROS generation; inhibition of lipid peroxidation and inflammatory pathways	Improved endothelial function and vascular redox balance	[243]
Green tea catechins (EGCG), turmeric extracts and polyphenol–protein complexes	Intestinal endocrine cell models (NCI-H716, STC-1)	Stimulate GLP-1 secretion through cAMP-dependent mechanisms and enteroendocrine cell activation	Reduce oxidative stress in intestinal environment; improve antioxidant capacity	Enhanced incretin secretion and satiety-related signalling	[218,224,225,226]
Polyphenol-rich foods (jabuticaba juice, citrus flavonoids, bilberry, anthocyanin-rich products)	Human nutritional intervention studies	Increase postprandial GLP-1 secretion and improve incretin response	Increase total antioxidant capacity; reduce oxidative biomarkers and lipid peroxidation	Improved postprandial glucose regulation and metabolic flexibility	[217,238,240]
Black soybean seed coat polyphenols	Rat models of metabolic dysfunction	Increase GLP-1 and cAMP signalling; enhance GLP-1-dependent vascular effects	Stimulate eNOS phosphorylation and NO production; improve antioxidant status in vascular and metabolic tissues	Improved insulin secretion, lipid profile and endothelial function	[215,216,241]
Curcumin	Animal and human metabolic models	Enhances GLP-1 secretion and improves incretin-mediated glucose regulation	Reduces ROS formation; improves mitochondrial function; suppresses inflammatory signalling	Improved glucose tolerance, reduced hepatic lipid accumulation and metabolic inflammation	[14,219,220,221,222,223]
Catechins (EGCG)	Cellular and experimental metabolic models	Stimulate GLP-1 release from enteroendocrine cells	Increase antioxidant capacity, reduce lipid peroxidation and improve mitochondrial stability	Improved glucose metabolism and cellular stress resistance	[225]
Natural DPP-4 inhibitors (myricetin, phlorizin and flavonoids)	Cellular and animal models	Inhibit DPP-4 activity, prolonging active GLP-1 availability	Reduce oxidative stress and inflammatory signalling; improve antioxidant defence	Improved glycaemic control and β-cell protection	[229,230,231,232]
Mulberry-derived compounds (DNJ, C3G, C3R)	Oxidative stress and insulin secretion models	Support GLP-1-related metabolic regulation	Increase glucokinase activity, ATP production and antioxidant protection of β-cells	Improved insulin secretion under oxidative stress conditions	[233,234,235]
Polyphenol-rich botanical preparations (yerba maté, Aronia, *Dendrobium*, grape pomace, grape seed extracts)	Experimental and nutritional models	Modulate GLP-1/GIP signalling and gut–metabolic communication	Improve antioxidant capacity, regulate microbiota-related oxidative pathways and reduce inflammation	Improved glucose homeostasis and metabolic resilience	[244,245,246,247,248]
Neuroprotective natural compounds (crocin, catechins, chalcone AN07)	Oxidative stress and neurodegeneration models	Modulate GLP-1-related neuroprotective signalling	Reduce ROS formation, lipid peroxidation and inflammatory activation	Neuroprotection and improved mitochondrial resilience	[249,250,251,252]
*Phyllanthus niruri* niosomal formulation	Obesity-induced hepatic steatosis in rats	Modulates GLP-1R-related signalling pathways	Improves hepatic mitochondrial function and antioxidant status	Reduced hepatic steatosis and improved metabolic parameters	[253]
Liraglutide + resveratrol combination	Experimental oxidative injury model	Combines pharmacological GLP-1R activation with polyphenol-mediated metabolic regulation	Enhances antioxidant defence, reduces apoptosis and inflammatory signalling	Improved tissue protection and reduced oxidative injury	[242]

Abbreviations: GLP-1—glucagon-like peptide-1; GLP-1R—glucagon-like peptide-1 receptor; GLP-1RAs—glucagon-like peptide-1 receptor agonists; GIP—glucose-dependent insulinotropic polypeptide; DPP-4—dipeptidyl peptidase-4; EGCG—epigallocatechin gallate; ROS—reactive oxygen species; eNOS—endothelial nitric oxide synthase; NO—nitric oxide; SIRT1—sirtuin-1; ATP—adenosine triphosphate; DNJ—1-deoxynojirimycin; C3G—cyanidin-3-glucoside; C3R—cyanidin-3-rutinoside.

**Table 3:** Plant-derived bioactive compounds and peptides modulating GLP-1 signalling: principal mechanisms and metabolic outcomes.

Bioactive Compound/Extract	Primary Mechanism Affecting GLP-1 Signalling	Major Metabolic and Redox Effects	Experimental Model	References
Curcumin (*Curcuma longa*)	Stimulates GLP-1 secretion; improves insulin signalling; suppresses oxidative stress	Improved glucose tolerance; reduced inflammation; enhanced antioxidant defence	Animal and human studies	[220,221]
*Fagonia cretica*	Natural TGR5 agonist promoting GLP-1 biosynthesis and secretion	Enhanced incretin response and glycaemic control	Enteroendocrine L-cells	[286]
Mangiferin (*Mangifera indica*)	Stimulates GLP-1 secretion and GLP-1 receptor signalling	Reduced fasting glucose; improved insulin sensitivity	Diabetic rats	[291]
*Momordica cochinchinensis* (Gac)	Taste receptor-mediated GLP-1 secretion	Improved glucose homeostasis and reduced inflammation	Diabetic mice	[287]
*Momordica charantia*	Bitter taste receptor activation inducing GLP-1 release	Enhanced insulin secretion and hypoglycaemic effects	Experimental models	[288]
Finger citron polysaccharides/*Achyranthes bidentata* polysaccharides	Gut microbiota modulation; increased SCFA production; FFAR-mediated GLP-1 secretion	Improved intestinal barrier, insulin secretion and glycaemic control	Diabetic mice	[264,265]
Yerba maté (*Ilex paraguariensis*)	Ferulic acid metabolism contributes to GLP-1 secretion	Improved incretin response and metabolic flexibility	Human studies	[244]
Jaboticaba peel extract	Polyphenol-mediated modulation of inflammatory pathways	Reduced postprandial glucose and inflammation	Human metabolic syndrome	[217]
Polyphenolic plant extracts	Enhancement of GLP-1RA signalling; antioxidant and endothelial protection	Reduced oxidative stress; improved cardiovascular metabolism	Heart failure/ metabolic models	[299]
Tulsi, Banyan and Jamun extracts	DPP-4 inhibition preserving endogenous GLP-1	Increased GLP-1 bioavailability and insulin secretion	In vitro	[292,293]
Fenugreek-derived peptides (*Trigonella foenum-graecum*)	Positive modulation of GLP-1 receptor signalling	Improved energy homeostasis and insulinotropic response	Experimental studies	[290]
*Coprinus comatus* mushroom	Increased GLP-1 and glutathione levels	Improved glycaemia and antioxidant capacity	Hyperglycaemic rats	[297]
*Elaeagnus umbellata* extracts	Interaction with GLP-1 receptor; inhibition of AGE–RAGE signalling	Reduced glycation and oxidative stress	In vitro*/*in silico	[300]
*Thymus praecox* extract	Restoration of β-cell function through GLP-1-associated pathways	Improved glucose homeostasis	Diabetic rats	[301]
Avocado paste extract	Increased GLP-1 mRNA expression	Improved satiety signalling and metabolic hormone profile	Wistar rats	[302]
Clove flower extract	Antioxidant activity indirectly supporting GLP-1-mediated tissue repair	Accelerated wound healing and reduced oxidative stress	Diabetic wound model	[303]
*Salicornia europaea*	Antioxidant phytochemicals indirectly supporting GLP-1 signalling	Improved antioxidant status and metabolic activity	Experimental studies	[304]
Lecithin–starch nanoparticles	Colon-targeted enhancement of GLP-1 secretion	Improved postprandial glucose regulation	Colon-targeted delivery system	[305]
Fruits of *Amomum villosum*	Antioxidant-mediated support of GLP-1 signalling	Reduced ROS and improved cellular resilience	In vitro	[306]

Abbreviations**:** GLP-1—glucagon-like peptide-1; GLP-1R—glucagon-like peptide-1 receptor; GLP-1RA—glucagon-like peptide-1 receptor agonist; TGR5—Takeda G protein-coupled receptor 5 (G protein-coupled bile acid receptor 1); DPP-4—dipeptidyl peptidase-4; SCFAs—short-chain fatty acids; FFAR2/FFAR3—free fatty acid receptors 2 and 3 (GPR43/GPR41); ROS—reactive oxygen species; GSH—reduced glutathione; AGE—advanced glycation end products; RAGE—Receptor for advanced glycation end products; STZ—streptozotocin; T2D—type 2 diabetes mellitus.

**Table 4:** Mitochondrial mechanisms regulating oxidative metabolism and insulin sensitivity in skeletal muscle.

Lp	Mechanisms	Definitions	Mitochondrial Effects	Metabolic Consequences	References
1	Lipid-induced mitochondrial stress	Accumulation of lipid intermediates disrupts mitochondrial homeostasis.	Increased ROS, impaired electron transport chain, reduced ATP synthesis.	Decreased insulin-stimulated glucose uptake; onset of insulin resistance.	[324,325]
2	Redox-sensitive insulin signalling	Oxidative stress modifies insulin-signalling proteins.	ROS alters IRS-1 and Akt phosphorylation, reducing mitochondrial efficiency.	Impaired GLUT4 translocation and reduced metabolic flexibility.	[326]
3	Exercise-induced mitochondrial resilience	Physical training enhances mitochondrial biogenesis and antioxidant capacity.	Increased PGC-1α, improved OXPHOS, stronger ROS buffering.	Improved insulin sensitivity and substrate oxidation.	[327]
4	Immune-metabolic mitochondrial regulation	Macrophage-derived cytokines modulate mitochondrial function in muscle fibres.	TNF-α/IL-6 increase ROS and reduce mitochondrial efficiency.	Inflammation-driven insulin resistance and reduced oxidative metabolism.	[328]
5	Mitochondrial remodelling after metabolic surgery	Weight-loss surgery induces structural and functional mitochondrial adaptations.	Enhanced β-oxidation, restored OXPHOS, reduced lipid overload.	Improved whole-body insulin sensitivity and metabolic recovery.	[329]

Abbreviations: ETC—electron transport chain; ROS—reactive oxygen species; OXPHOS—oxidative phosphorylation; IRS-1—insulin receptor substrate-1; PGC-1α—peroxisome proliferator-activated receptor gamma coactivator-1 alpha; TNF-α—tumour necrosis factor-alpha; IL-6—interleukin-6; GLUT4—glucose transporter type 4; ATP—adenosine triphosphate.

## Data Availability

No new data were created or analysed in this study. The data presented in this study are available within the article.

## Abbreviations

ABP—*Achyranthes bidentata* polysaccharide; AC—adenylyl cyclase; AGE—advanced glycation end products; Akt—protein kinase B; ALT—alanine aminotransferase; AMPK—AMP-activated protein kinase; AMP—adenosine monophosphate; APRIL—a proliferation-inducing ligand; ARE—antioxidant response element; AST—aspartate aminotransferase; ATP—adenosine triphosphate; BAFF—B-cell activating factor; cAMP—cyclic adenosine monophosphate; CAT—catalase; C3G—cyanidin-3-glucoside; C3R—cyanidin-3-rutinoside; COX-2—cyclooxygenase-2; CREB—cAMP response element-binding protein; CRP—C-reactive protein; DAG—diacylglycerol; DPP-4—dipeptidyl peptidase-4; DNJ—1-deoxynojirimycin; EGCG—epigallocatechin gallate; eNOS—endothelial nitric oxide synthase; EPCs—endothelial progenitor cells; Epac—exchange protein directly activated by cAMP; EPAC2—exchange protein directly activated by cAMP 2; ETC—electron transport chain; FFAR2—free fatty acid receptor 2 (GPR43); FFAR3—free fatty acid receptor 3 (GPR41); FDX1—ferredoxin 1; FGF-19—fibroblast growth factor 19; FGF-21—fibroblast growth factor 21; FHRE—forkhead response element; FoxO—Forkhead box O; FOXO3a—forkhead box O3a; FXR—farnesoid X receptor; Gαgust—gustducin alpha subunit; Gαs—stimulatory G protein alpha subunit; Gβγ—G protein beta-gamma subunits; GCG—glucagon gene; GCLC—glutamate–cysteine ligase catalytic subunit; GCLM—glutamate–cysteine ligase modifier subunit; GIP—glucose-dependent insulinotropic polypeptide; GLP-1—glucagon-like peptide-1; GLP-1R—glucagon-like peptide-1 receptor; GLP-1RA—glucagon-like peptide-1 receptor agonist; GPx—glutathione peroxidase; GPR41—G-protein-coupled receptor 41; GPR43—G-protein-coupled receptor 43; Gq—G protein alpha q subunit; GSH—reduced glutathione; GSK-3β—glycogen synthase kinase-3 beta; GST—glutathione S-transferase; HFpEF—heart failure with preserved ejection fraction; HMGB1—high-mobility group box 1; HO-1—heme oxygenase-1; Iba1—ionised calcium-binding adaptor molecule 1; ICAM-1—intercellular adhesion molecule-1; IL—interleukin; IL-1β—interleukin-1 beta; IL-6—interleukin-6; iNOS—inducible nitric oxide synthase; IP3—inositol 1,4,5-trisphosphate; IRS-1—insulin receptor substrate-1; JAK—Janus kinase; JNK—c-Jun N-terminal kinase; Keap1—Kelch-like ECH-associated protein 1; LC3—microtubule-associated protein 1 light chain 3; LC3β—microtubule-associated protein 1 light chain 3 beta; LOX-1—lectin-like oxidised low-density lipoprotein receptor-1; MAPK—mitogen-activated protein kinase; MASLD—metabolic dysfunction-associated steatotic liver disease; MCP-1—monocyte chemoattractant protein-1 (CCL2); MDA—malondialdehyde; mtDNA—mitochondrial DNA; mtROS—mitochondrial reactive oxygen species; NADPH—nicotinamide adenine dinucleotide phosphate; NF-κB—nuclear factor kappa B; NLRP3—NOD-, LRR- and pyrin domain-containing protein 3; NO—nitric oxide; NOX—NADPH oxidase; NQO1—NAD(P)H quinone dehydrogenase 1; NRF1—nuclear respiratory factor 1; Nrf2—nuclear factor erythroid 2-related factor 2; 8-iso-PGF2α—8-isoprostaglandin F2α; OXPHOS—oxidative phosphorylation; ox-LDL—oxidised low-density lipoprotein; PCOS—polycystic ovary syndrome; PGC-1α—peroxisome proliferator-activated receptor gamma coactivator 1-alpha; PI3K—phosphoinositide 3-kinase; PKA—protein kinase A; PKC—protein kinase C; PLC—phospholipase C; PLCβ—phospholipase C beta; PLCβ2—phospholipase C beta 2; PLCε—phospholipase C epsilon; PRDX—peroxiredoxin; PVAT—perivascular adipose tissue; RAGE—receptor for advanced glycation end products; Rap1—Ras-related protein 1; RNS—reactive nitrogen species; ROS—reactive oxygen species; SAT—subcutaneous adipose tissue; SCFAs—short-chain fatty acids; SGLT2i—sodium–glucose cotransporter-2 inhibitors; SIRT—sirtuin; SIRT1—sirtuin 1; SNARE—soluble N-ethylmaleimide-sensitive factor attachment protein receptor; SOD—superoxide dismutase; STAT3—signal transducer and activator of transcription 3; STZ—streptozotocin; T2D—type 2 diabetes mellitus; TBARS—2-thiobarbituric acid-reactive substances; TCF4—T-cell factor 4; TFAM—mitochondrial transcription factor A; TGR5—Takeda G-protein-coupled receptor 5 (G protein-coupled bile acid receptor 1); TNF-α—tumour necrosis factor-alpha; TXN—thioredoxin; UCP2—uncoupling protein 2; VAT—visceral adipose tissue; VCAM-1—vascular cell adhesion molecule-1; Wnt—wingless/integrated; β-Klotho—beta-Klotho.
